# Differential Investment Strategies in Leaf Economic Traits Across Climate Regions Worldwide

**DOI:** 10.3389/fpls.2022.798035

**Published:** 2022-03-04

**Authors:** Liang Ren, Yongmei Huang, Yingping Pan, Xiang Xiang, Jiaxuan Huo, Dehui Meng, Yuanyuan Wang, Cheng Yu

**Affiliations:** ^1^State Key Laboratory of Earth Surface Processes and Resource Ecology, Faculty of Geographical Science, Beijing Normal University, Beijing, China; ^2^School of Natural Resources, Faculty of Geographical Science, Beijing Normal University, Beijing, China

**Keywords:** plant strategies, leaf economics spectrum (LES), functional traits, trade-offs, Köppen–Geiger climate classification, allometry, trait variation

## Abstract

The leaf economics spectrum (LES) is the leading theory of plant ecological strategies based on functional traits, which explains the trade-off between dry matter investment in leaf structure and the potential rate of resource return, revealing general patterns of leaf economic traits investment for different plant growth types, functional types, or biomes. Prior work has revealed the moderating role of different environmental factors on the LES, but whether the leaf trait bivariate relationships are shifted across climate regions or across continental scales requires further verification. Here we use the Köppen–Geiger climate classification, a very widely used and robust criterion, as a basis for classifying climate regions to explore climatic differences in leaf trait relationships. We compiled five leaf economic traits from a global dataset, including leaf dry matter content (LDMC), specific leaf area (SLA), photosynthesis per unit of leaf dry mass (A_mass_), leaf nitrogen concentration (N_mass_), and leaf phosphorus concentration (P_mass_). Moreover, we primarily used the standardized major axis (SMA) analysis to establish leaf trait bivariate relationships and to explore differences in trait relationships across climate regions as well as intercontinental differences within the same climate type. Leaf trait relationships were significantly correlated across almost all subgroups (*P* < 0.001). However, there was no common slope among different climate zones or climate types and the slopes of the groups fluctuated sharply up and down from the global estimates. The range of variation in the SMA slope of each leaf relationship was as follows: LDMC–SLA relationships (from −0.84 to −0.41); A_mass_–SLA relationships (from 0.83 to 1.97); A_mass_–N_mass_ relationships (from 1.33 to 2.25); N_mass_–P_mass_ relationships (from 0.57 to 1.02). In addition, there was significant slope heterogeneity among continents within the Steppe climate (BS) or the Temperate humid climate (Cf). The shifts of leaf trait relationships in different climate regions provide evidence for environmentally driven differential plant investment in leaf economic traits. Understanding these differences helps to better calibrate various plant-climate models and reminds us that smaller-scale studies may need to be carefully compared with global studies.

## Introduction

It is well-known that plant functional traits have the potential to explain species’ adaption strategies and the response of plants to environments (Westoby and Wright, 2006). In most previous studies, leaf traits are considered to be sensitive and important indicators of environmental changes and particularly useful in explaining how plants are responding to the current climate change of great concern (Garnier et al., 2001; Poorter et al., 2009; Salazar Zarzosa et al., 2021). Quantifying the relationship between leaf functional traits and climate is the key to explaining what traits make plants suitable for living in specific climatic regions and have the potential to predict the response of communities and ecosystems to future climate change (Heilmeier, 2019). Indeed, plant functional traits tend not to vary independently (Reich et al., 1997, 2003; Wright et al., 2001, 2004), which shows covariation or coordination caused by the allocation of limited resources to balance different needs (Candeias and Fraterrigo, 2020). The bivariate trait relationships are not invariant but converge over a wide geographical range, as summarized by Wright et al. (2004) for the “leaf economics spectrum” (LES), which reflects a trade-off between the cost of leaf structure and the rate of resource return. Moreover, many subsequent studies have verified the existence of this relationship in additional ecosystems or under extreme climate conditions (He et al., 2006; Freschet et al., 2010; Fynn et al., 2011). Based on the framework of “economics spectrum,” wood (Chave et al., 2009), root (Roumet et al., 2016), seed (Saatkamp et al., 2019), flower (Roddy et al., 2021), and whole plant economics spectrums (Reich, 2014; Díaz et al., 2016) have been proposed in recent years.

Although the LES of vascular plants on a global scale has been widely accepted, this is not sufficient for us to understand the contribution of trait variability or plasticity to the differential adaptation of plants to specific climatic regions (Cui et al., 2020). Climatic factors such as temperature and precipitation are important predictors of some leaf functional traits (Moles et al., 2014; Volaire, 2018), which means that they have the potential to explain plant adjustments to leaf trait relationships (Wright et al., 2005b; Blonder et al., 2013). At a global scale, leaf N and P decrease with increasing average temperature and with nearness to the equator (Reich and Oleksyn, 2004). The relationship between leaf lifespan (LL) and leaf mass per area (LMA) tends to be shifted with climate, showing a flatter slope with increasing site MAT, VPD, PET, and irradiance (Wright et al., 2004, 2005b). These trait relationships on environmental gradients are considered as secondary trade-offs between the actual plant response to abiotic or biotic factors (Suding et al., 2003; Gross et al., 2007). However, it is not clear to us whether the broad global climate gradient drives secondary trade-offs of leaf functional traits in different patterns. Previous studies have made us aware of the importance of climate effects on leaf bivariate relationships, but it is difficult for a single climate factor to explain variation in multiple traits, which makes the trait-climate relationship complex and confusing (Moles et al., 2014; Meng et al., 2017). This complexity is one of the critical factors limiting the development of many earth system models (Bodegom et al., 2012; Verheijen et al., 2015). For example, modelers often rely on strong correlations between traits shown in large-scale studies to assign global or regional model parameter values, but heterogeneity in environmental factors such as climate may increase the uncertainty of prediction (Wang et al., 2012, 2017b; Kovenock and Swann, 2018). Hence, understanding how leaf trait relationships behave in different climate regions can enable better calibration of earth system models and is crucial for predicting how plants will respond to future climate change.

**FIGURE 1:**
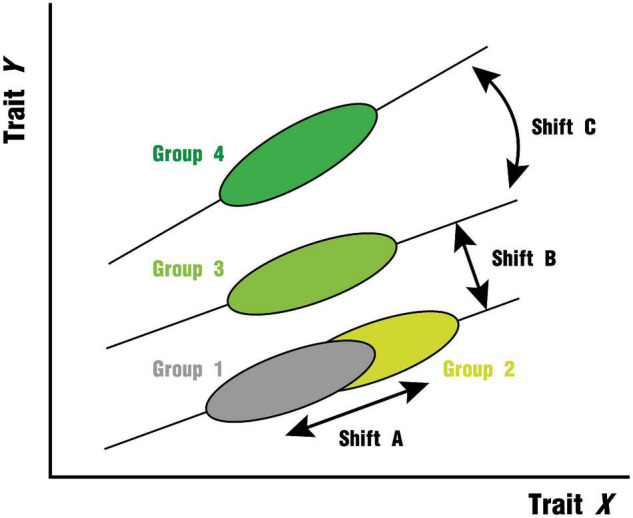
Hypotheses for shifts in leaf trait relationships based on allometric relationships (Adapted from Wright et al., 2001). (1) Shift A indicates that the trait pairs of the two groups are on a common axis without heterogeneity in slope or intercept; (2) Shift B indicates that the slope of the trait relationship is similar between the two groups, but the difference in intercept is significant; (3) Shift C indicates that the slope of the trait relationship between the two groups is heterogeneous. Modulated by climatic resources (e.g., precipitation, temperature), shifts B and C are both possible. Furthermore, we cannot exclude the influence of biogeographic differences inherent in the region (e.g., species composition, evolutionary history) may have an effect.

The leaf economic traits are closely related to each other and the relationship between trait x and trait y can be expressed by the equation: *y* = *cx*^b^ (Reich et al., 1997; Wright et al., 2004; Warton et al., 2006). After the trait is logarithmically transformed, the equation is: log*y* = *b*log*x* + *a*,*a* = log*c*, where a and b represent the intercept and slope. Along the environmental gradient, the leaf trait relationships of different plant groups may differ in slope or elevation (i.e., intercept when the slope is the same) due to differences in resource allocation (Wright et al., 2001; Heberling and Fridley, 2012), or they may be strongly constrained by intrinsic factors such as the genetic background and elemental metabolism of the plants to exhibit functional convergence and thus be distributed along a common axis (Figure 1). The test for slope heterogeneity (Figure 1, Shift C) is the prior step in the multiple group comparisons of bivariate relationships, and when a common slope exists for all subgroups, we can further test for intercept heterogeneity (Figure 1, Shift B). The slope of the trait relationship is informative and important because it can be used as an indicator of plant resource capture strategies on environmental gradients (Leishman et al., 2007; Heberling and Fridley, 2012; Guo et al., 2020). If the slope of the fit is not significantly different across groups, it suggests that the trait relationship is relatively robust across environments (Cui et al., 2020). In this study, we compiled a leaf functional trait dataset covering 82,957 observations of 7,523 species distributed over six continents with the help of data sets provided by TRY Plant Trait Database.^1^ Each observation was matched to a climate zone and climate type corresponding to the Köppen–Geiger climate classification (Peel et al., 2007). The Köppen–Geiger climate classification is a highly advantageous classification scheme that combines monthly and annual average temperature and precipitation data, can be used to predict biome distributions, considers thresholds for plant sensitivity, and facilitates the construction of bioclimatic models and the analysis of vegetation response under future climate change (Mahlstein et al., 2013). There have been many advances in the effects of single climatic factors on traits and trait relationships (Reich and Oleksyn, 2004; Wright et al., 2005b; Ordoñez et al., 2009), but realistic vegetation distribution areas are spatially compounded by multiple factors, and the role of these factors may be difficult to be stripped away. Then the classification of climate regions would be a new perspective to assess the generality of global leaf trait bivariate relationships. Furthermore, since many climate types are widely dispersed across continents, we consider different continents within the same climate type as a smaller scale where bivariate relationships are more constrained by non-climatic biogeographic constraints (e.g., differences in dominant species, differences in evolutionarily distinct floras) (Heberling and Fridley, 2012). This extended comparison may help explain potential differences in trait relationships between plants of different functional groups or geographic locations due to specific evolutionary history and environmental conditions (Tian et al., 2018). Here, we aimed to compare the relationships between key leaf traits across different climate regions and attempt to compare intercontinental differences within the same climate type. These two tasks correspond to the following two important sets of questions:

(1) Are the leaf trait relationships in different climate regions consistent with the global pattern? If not, do these relationships reflect differential investment in traits?

(2) Are leaf trait relationships within different continents along a common axis when controlling for differences in climate type? Are the slope estimates for these regions consistent with global estimates?

## Materials and Methods

### Leaf Economic Traits and Trait Relationships

For the selection of leaf traits, we considered five ecologically important and closely linked leaf functional traits: leaf dry matter content (LDMC), specific leaf area (SLA), leaf photosynthesis per leaf dry mass (A_mass_), leaf nitrogen concentration (N_mass_), and leaf phosphorus concentration (P_mass_). Specifically, LDMC reflects the investment made in leaf tissue construction (Shipley et al., 2006; Hu et al., 2015). SLA represents the light-interception area per unit leaf mass (Wright et al., 2004). A_mass_ refers to the maximum photosynthetic rate measured under high light, ample soil moisture, and ambient CO_2_, which is mainly influenced by the stomatal conductance as well as the diffusion resistance and diffusion path of CO_2_ within the leaf (Wright et al., 2004; Zhang et al., 2015). N_mass_ and P_mass_ are mass-based expressions of leaf nitrogen content and leaf phosphorus content (Perez-Harguindeguy et al., 2013; Table 1). Some scholars also analyzed with area-based leaf nitrogen content and leaf phosphorus content (Lloyd et al., 2013; Osnas et al., 2013), but the trait relationships derived from area-based expression were not well-suited to be understood in terms of plant growth and economics (Westoby et al., 2013). N_mass_ can be seen as the potential photosynthetic carboxylation capacity because proteins of the Calvin cycle and thylakoids represent the majority of leaf nitrogen (Field and Mooney, 1986; Evans, 1989), while P_mass_ is critical for photosynthesis and metabolic processes (Reich et al., 2009). Moreover, N_mass_ and P_mass_ are closely related and often together reflect the nutrient limitation of the habitat and the influence of climatic factors such as temperature and moisture (Reich and Oleksyn, 2004; Han et al., 2005).

**TABLE 1:** List of leaf traits selected for this study, their abbreviations, functional significance, and related references.

Traits	Abbreviations	Unit	Functional significance and relevant literature
Leaf dry matter content per leaf water-saturated mass	LDMC	g g^–1^	Growth, competitive ability, stress tolerance (Wilson et al., 1999; Cornelissen et al., 2003; Kleyer et al., 2008)
Leaf area per leaf dry mass	SLA	mm^2^ mg^–1^	Growth, resource acquisition, and use (Wilson et al., 1999; Wright et al., 2004; Díaz et al., 2016)
Photosynthesis per leaf dry mass	A_mass_	μmol g^–1^s^–1^	Resource acquisition and use (Wright et al., 2004; Zhang et al., 2015)
Leaf nitrogen content per dry mass	N_mass_	mg g^–1^	Nutrient conservation, decomposition (Perez-Harguindeguy et al., 2013; de la Riva et al., 2019)
Leaf phosphorus content per dry mass	P_mass_	mg g^–1^	Nutrient conservation, decomposition (Perez-Harguindeguy et al., 2013; de la Riva et al., 2019)

The correlation of these leaf traits makes them tightly connected within a dimension, and there are specific causal relationships behind these links (Shipley et al., 2006). The bivariate relationships within the LES dimension are all related to resource acquisition and nutrient conservation strategies and can reflect whether the plant prefers a fast or slow growth strategy. To compare the effects of environmental gradients, we selected a subset of LES relationships, which involve structural, chemical, and physiological aspects of leaves. The details are as follows: (1) LDMC–SLA relationship (fundamental trade-off between leaf structural traits); (2) A_mass_–SLA relationship (allometry between leaf physiological and structural traits); (3) A_mass_–N_mass_ relationship (allometry between leaf physiological and chemical traits); (4) N_mass_–P_mass_ relationship (allometry between leaf chemical traits).

### Data Description

In this study, 82,957 observations of 7,523 plant species worldwide were collected from the TRY Plant Trait Database. In addition to the original dataset of leaf economic spectrum (GLOPNET), 69 other datasets were involved in compiling the data (Cornelissen et al., 1996; Baruch and Goldstein, 1999; Shipley, 2002; Adler et al., 2004; Wright et al., 2004; Louault et al., 2005; Michaletz and Johnson, 2006; Craven et al., 2007; Swaine, 2007; Montgomery and Givnish, 2008; Craine et al., 2009; Kattge et al., 2009; van de Weg et al., 2009; Freschet et al., 2010; Messier et al., 2010; Ordoñez et al., 2010; Powers and Tiffin, 2010; Wright et al., 2010; Butterfield and Briggs, 2011; Campetella et al., 2011; Laughlin et al., 2011; Prentice et al., 2011; Swenson et al., 2011; Han et al., 2012; Rolo et al., 2012; van de Weg et al., 2012; Vergutz et al., 2012; Auger and Shipley, 2013; Boucher et al., 2013; Chen et al., 2013; Dahlin et al., 2013; Kichenin et al., 2013; Lukes et al., 2013; Martinez-Garza et al., 2013; Joseph et al., 2014; van der Plas and Olff, 2014; Seymour et al., 2014; Siefert et al., 2014; Smith et al., 2014; Takkis, 2014; Walker et al., 2014; La Pierre and Smith, 2015; Li et al., 2015; Maire et al., 2015; Blonder et al., 2016; Gos et al., 2016; Lhotsky et al., 2016; Petter et al., 2016; Herz et al., 2017; Wang et al., 2017a; Chacon-Madrigal et al., 2018; Dalke et al., 2018; Li and Shipley, 2018; Miller et al., 2018; Buchanan et al., 2019; Kattenborn et al., 2019; Sharpe and Solano, unpublished data). A detailed list of data contributions can be found in Supplementary Table 1.

During data retrieval, data sets containing two or more target traits at the same time will be initially accepted. Data from artificially controlled experiments rather than under natural conditions or without a location will be excluded to ensure that trait data can be matched with actual site environmental data. In addition, we used a metric of the risk of error (z-scores) provided by TRY to remove outliers with obvious errors and potentially high risk (Kattge et al., 2011). Trait records with z-scores >4 are those with distances greater than >4 standard deviations from the mean of the species, genus, family, or higher-rank taxonomic group (Díaz et al., 2016), and they were directly excluded because they have a very small probability and are likely to have some problems, such as non-standardized methods, wrong unit, measurements under very uncommon conditions (Kattge et al., 2011). In our dataset, trait values were extracted from each observation for the individuals of each species, and each site may contain observations of one or hundreds of species, so we were able to calculate site means of traits for sites that contain at least four species. It is worth mentioning that the sample size of the site means is much smaller than that of individual values, so we compared the two methods in the global analysis and the climate zone analysis, while our further analysis used only the individual values of each trait (Table 2).

**TABLE 2:** Summary of leaf economic traits based on individual species values and site mean values.

Traits	N	Mean	SD	CV (%)	Minimum	Maximum
**Individual trait values**						
SLA (mm^2^ mg^–1^)	77,210	20.01	12.39	61.9	1.09	148.08
LDMC (g g^–1^)	67,111	0.31	0.10	33.0	0.05	0.89
A_mass_ (μmol g^–1^s^–1^)	6,246	0.15	0.11	76.2	0.01	1.28
N_mass_ (mg g^–1^)	23,470	19.80	8.42	42.6	0.60	79.09
P_mass_ (mg g^–1^)	15,757	1.47	1.03	69.8	0.09	9.59
**Site mean trait values**						
SLA (mm^2^ mg^–1^)	690	16.29	8.17	56.2	1.91	71.41
LDMC (g g^–1^)	401	0.30	0.08	26.0	0.12	0.55
A_mass_ (μmol g^–1^s^–1^)	143	0.14	0.08	55.6	0.03	0.43
N_mass_ (mg g^–1^)	460	19.64	6.58	33.5	0.88	42.12
P_mass_ (mg g^–1^)	360	1.30	0.68	52.7	0.15	4.19

**FIGURE 2:**
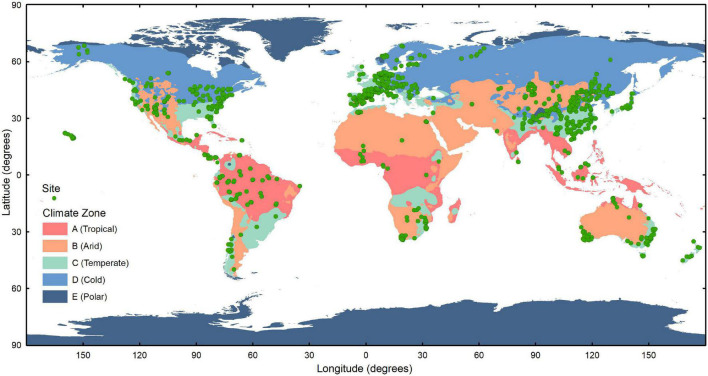
Distribution of the sites to which the leaf traits belong in different climate zones of the world. The climate zones are divided into A (Tropical), B (Arid), C (Temperate), D (Cold), and E (Polar) zones with reference to the Köppen–Geiger system (Peel et al., 2007).

Using the location of each trait record, we matched the continent to which it belonged and the corresponding climate zone and climate type in the Köppen–Geiger climate classification (Peel et al., 2007). Our dataset covers most of the spatial distribution of vegetation and is large enough to support the analysis of different climate regions on six continents, with the exception of Antarctica (Figure 2 and Supplementary Figure 1). Based on the criteria of this climate classification, we have divided the climate region first into five climate zones, which are further divided into 12 climate types (Table 3). In particular, only leaf trait data for the Tundra climate type (ET) was included within the Polar climate zone because of the scarcity of observations within the Frost climate type (EF).

**TABLE 3:** Codes defined in Köppen–Geiger climate classification and their meanings (Adapted from Peel et al., 2007).

Climate zones (prefix)	Climate type (suffix)
A (Tropical)	f (Rainforest)
	m (Monsoon)
	w (Savannah)
B (Arid)	W (Desert)
	S (Steppe)
C (Temperate)	s (Dry Summer)
	w (Dry Winter)
	f (Without dry season)
D (Cold)	s (Dry Summer)
	w (Dry Winter)
	f (Without dry season)
E (Polar)	T (Tundra)
	F (Frost)

### Statistical Analysis

All leaf trait data were log_10_-transformed and the transformed data were approximately normally distributed. Bivariate relationships for leaf traits were fitted by standardized major axis (SMA) regression because it takes into account the concurrent errors in the x and y axes (Wright et al., 2005b; Heberling and Fridley, 2012; Cui et al., 2020). SMA regression and further inference on the fitted lines can be done in R 4.0.4 using the “smart” package version 3.0 (Warton et al., 2012; R Core Team, 2020). First, the best estimate of the slope and intercept was fitted by the SMA function based on all observations, and the fitted line represents the convergence of the global plant function on one axis. Moreover, the SMA function can test the slope or intercept heterogeneity of several fitted lines by specifying parameters, which include constructing confidence intervals for a common slope or intercept and using a one-sample test to examine the common slope or intercept between fitted lines. This allows us to check if there is a common slope between different groups (typically α = 0.05). If the slopes are heterogeneous (*P* < 0.05), we perform multiple comparisons between groups and use adjusted *P*-values to control family-wise error rates in a conservative way. Additionally, SMA allows us to compare the slope of each group with the results of global estimation in order to analyze whether a globally estimated parameter applies to groups divided by different climate zones or other types. If the slopes are homogeneous (*P* > 0.05), we further compare the variation of intercepts.

## Results

### Global Patterns of Leaf Economic Trait Relationships

Globally, variation in five leaf economic traits was different, and the mean values of the traits at the species level and averaged by site were very close (Table 2). LDMC was the least variable at both the species level and the site level (CV = 33%, CV = 26%, respectively). P_mass_ showed the greatest variation at the species level (CV = 69.8%), while SLA showed the greatest variation at the site level (CV = 56.2%). Moreover, there was a significant correlation between the five leaf traits (*P* < 0.001). The relationships we described at the species level for leaf economic traits (except for LDMC–SLA relationship) with larger sample sizes were very consistent with those described in previous global dataset GLOPNET (Wright et al., 2004), and these relationships were validated at the site level (Table 4). Across species, SLA was negatively correlated with LDMC (*R*^2^ = 0.12, *P* < 0.001), but positively correlated with A_mass_ (*R*^2^ = 0.45, *P* < 0.001). N_mass_ showed a significant positive correlation with both A_mass_ and P_mass_ (*R*^2^ = 0.41, *P* < 0.001; *R*^2^ = 0.62, *P* < 0.001). With the exception of the A_mass_–SLA relationship, the leaf trait relationship showed a higher *R*^2^ at the site level.

**TABLE 4:** Comparison of leaf trait relationships on a global scale.

Trait relationships	Species			Sites			GLOPNET dataset (Wright et al., 2004)		
	Slope (95% CIs)	*R* ^2^	N	Slope (95% CIs)	*R* ^2^	N	Slope (95% CIs)	*R* ^2^	N
LDMC–SLA	−0.60 (−0.60, −0.59)	0.12	66,918	−0.55 (−0.59, −0.50)	0.35	381	−	−	−
A_mass_–SLA	1.25 (1.23, 1.27)	0.45	6,107	1.32 (1.13, 1.54)	0.33	113	1.32 (1.26, 1.40)	0.50	764
A_mass_–N_mass_	1.73 (1.69, 1.77)	0.41	4,486	1.59 (1.40, 1.80)	0.62	96	1.72 (1.63, 1.81)	0.53	712
N_mass_–P_mass_	0.66 (0.65, 0.67)	0.27	15,589	0.74 (0.69, 0.79)	0.59	348	0.66 (0.64, 0.69)	0.72	745

*SMA slope and 95% confidence interval (CI), coefficient of determination (R^2^), and sample sizes are given for each of the three data sets: (1) correlations based on individual species trait values (“Species”); (2) correlations based on site mean trait values (“Sites”); (3) correlations based on species trait values in GLOPNET (Wright et al., 2004).*

### Comparison of Standardized Major Axis Slopes Among Different Climate Regions

**FIGURE 3:**
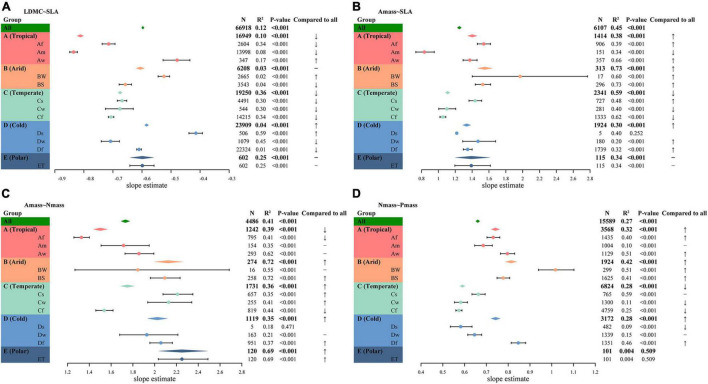
Leaf trait relationships within different climate regions. **(A)** LDMC–SLA relationship. **(B)** Amass–SLA relationship. **(C)** A_mass_–N_mass_ relationship. **(D)** N_mass_–P_mass_ relationship. The diamond represents the fitted standardized major axis (SMA) slope estimate for all observations globally or within a subgroup. The points with error lines give the slope estimates and 95% confidence intervals for each climate type. A “↑” indicates the slope is significantly higher than the global estimate (*P* < 0.05), “↓” indicates the slope is significantly lower than the global estimate (*P* < 0.05), “−” indicates the slope is not significantly different from the global estimate (*P* > 0.05). In addition, the sample sizes, coefficients of determination (*R*^2^), and *P*-values for all SMA fits are given.

At the species level, shifts in leaf trait relationships were substantial in some climate regions (Figure 3), which was also reflected at the site level, although the slope variation range at the site level was narrower (Supplementary Table 3). Because species-level trait relationships were directly related to the species’ resource acquisition and utilization strategies, here we only showed analyses based on individual species trait data.

#### Leaf Dry Matter Content–Specific Leaf Area Relationships

There was a significant negative correlation between LDMC and SLA in all climate regions (*P* < 0.001). At the species level, there was no common slope among the different climate zones (*P* ≪ 0.001), although SMA slopes within Arid climate (B) and Polar climate (E) were not significantly different from the global estimate (Figure 3A and Supplementary Table 3). SMA slope fitted within the Tropical climate (A) showed the lowest slope of the several climate zones and decreased from the global estimate of −0.6 to −0.81. The SMA slope within the Cold climate (D) was the flattest, significantly less negative than the slope within the Temperate climate (C) and the Tropical climate (−0.58 vs. −0.68, *P* < 0.001; −0.58 vs. −0.81, *P* < 0.001), but not significantly different from the slope within the Arid climate or the Polar climate (both *P* > 0.05). Also, the SMA slopes were heterogeneous among the different climate types (*P* ≪ 0.001). The LDMC-SLA trade-offs varied considerably among climate types, ranging from −0.84 for the Am climate to −0.41 for the Ds climate.

#### Photosynthesis Per Leaf Dry Mass–Specific Leaf Area Relationships

Higher A_mass_ was often associated with higher SLA across species (Figure 3B). The SMA slopes were detected to be heterogeneous among different climate zones (*P* ≪ 0.001) and only the Polar climate shared a common slope with global estimates. The Arid climate held the steepest SMA slope of 1.55, while the Temperate climate had a slope of only 1.11. Moreover, the slope estimates were not homogeneous within different climate types, with the Am climate slope decreasing from a global estimate of 1.25–0.83, while the slope within the BW climate type was as high as 1.97. With the exception of the ET climate, the SMA slopes of the other climates were significantly different from the global estimate. In addition, smaller sample sizes may lead to increased errors in estimation and even decoupling of leaf trait relationships, such as those shown in Figure 3B for the BW and the Ds climate types.

#### Photosynthesis Per Leaf Dry Mass–Leaf Nitrogen Concentration Relationships

A_mass_ and N_mass_ were positively correlated within each climate region, except for the Ds climate (Figure 3C). This relationship had a roughly increasing slope with decreasing heat in different climate zones, except for a fluctuation in the Arid climate. Compared to the global estimates, there was no significant shift in slope within the Temperate climate, but the slopes were significantly shifted within the remaining climate zones. The SMA slopes among different climate zones and climate types were heterogeneous (both *P* ≪ 0.001). The SMA slope of the Af climate was the flattest, while the slope of the ET was the steepest.

#### Leaf Nitrogen Concentration–Leaf Phosphorus Concentration Relationships

N_mass_ and P_mass_ were positively correlated within each climate region, except for the Polar climate (Figure 3D). Although the differences between the Tropical, Cold, and Polar climates were small, the slope shift between the other climates was significant (Supplementary Table 4). Within the Temperate climate, the SMA slope was significantly lower than the global estimate of 0.66, while within the remaining three climate zones the slope was significantly higher than the global estimate, with the highest slope in the Arid climate reaching 0.81. The slopes fitted within the vast majority of climate types were heterogeneous and deviated significantly from the global estimates (Supplementary Table 5).

### Comparison of Standardized Major Axis Slopes Among Different Continents Within a Common Climate Type

To explore the robustness of leaf trait relationships across continents within the same climate type, we selected the BS climate and the Cf climate, which had relatively adequate sample sizes and were widespread across six continents, for further analysis.

#### Intercontinental Differences of Leaf Trait Relationship Within the Steppe Climate

**FIGURE 4:**
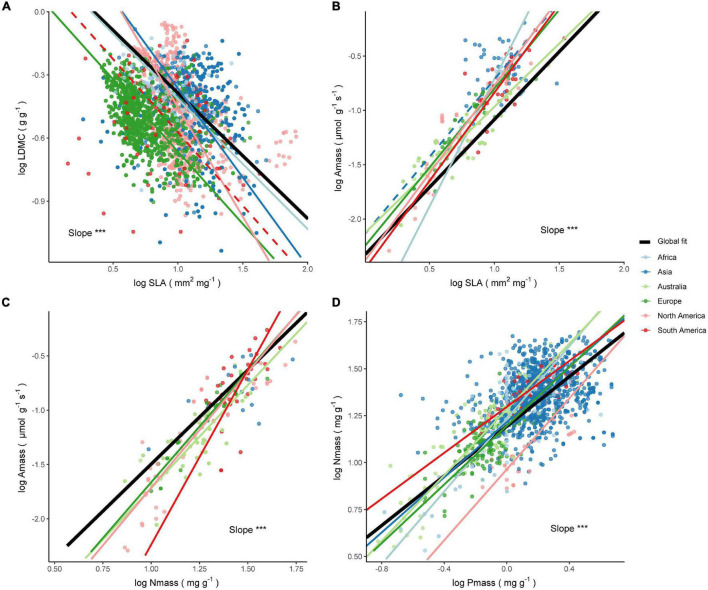
Relationships between leaf economic traits across species within the BS climate. The significance test results of the slope heterogeneity among the fitted lines are shown (***P < 0.001). The solid line is a significant fit to the standardized major axis (SMA) regression within a continent (*P* < 0.05), while insignificant relationships are shown as dashed lines (*P* > 0.05). Black solid lines are SMA regression lines for all species on a global scale. The respective slopes, sample sizes, and coefficients of determination of the SMA regressions are as follows (all *P* < 0.001 unless noted): **(A)** LDMC–SLA relationship. Africa: *b* = −0.62, *n* = 64, *R*^2^ = 0.06; Asia: *b* = −0.84, *n* = 1048, *R*^2^ = 0.03, *P* = 0.04; Europe: *b* = −0.69, *n* = 1623, *R*^2^ = 0.18; North America: *b* = −1.03, *n* = 740, *R*^2^ = 0.22; South America: *b* = −0.7, *n* = 68, *R*^2^ = 0.02, *P* = 0.29. **(B)** A_mass_–SLA relationship. Africa: *b* = 2.35, *n* = 14, *R*^2^ = 0.95; Asia: *b* = 1.43, *n* = 42, *R*^2^ = 0.03, *P* = 0.27; Australia: *b* = 1.16, *n* = 122, *R*^2^ = 0.71; Europe: *b* = 1.45, *n* = 46, *R*^2^ = 0.86; North America: *b* = 1.62, *n* = 42, *R*^2^ = 0.83; South America: *b* = 1.62, *n* = 30, *R*^2^ = 0.46. **(C)** A_mass_–N_mass_ relationship. Asia: *b* = 1.42, *n* = 16, *R*^2^ = 0.39, *P* = 0.01; Australia: *b* = 1.89, *n* = 123, *R*^2^ = 0.58; Europe: *b* = 2.05, *n* = 40, *R*^2^ = 0.89; North America: *b* = 2.12, *n* = 47, *R*^2^ = 0.77; South America: *b* = 3.22, *n* = 32, *R*^2^ = 0.68. **(D)** N_mass_–P_mass_ relationship. Africa: *b* = 0.97, *n* = 98, *R*^2^ = 0.62; Asia: *b* = 0.73, *n* = 1128, *R*^2^ = 0.11; Australia: *b* = 0.88, *n* = 190, *R*^2^ = 0.69; Europe: *b* = 0.78, *n* = 123, *R*^2^ = 0.53; North America: *b* = 0.94, *n* = 37, *R*^2^ = 0.45; South America: *b* = 0.61, *n* = 49, *R*^2^ = 0.6.

The four trait relationships did not share a common slope across continents within the BS climate (all *P* < 0.01), and many comparisons between continents exhibited slope heterogeneity (Supplementary Table 6). LDMC within different continents was negatively correlated with SLA, except for the decoupling of the relationship in South America. The LDMC–SLA relationship within Asia, Europe, and North America exhibited a steeper slope and differed significantly from the global estimate, while the slope estimate for Africa didn’t differ significantly from the estimated value of 0.6 (Figure 4A and Supplementary Table 8). The correlation of the A_mass_–SLA relationship in Asia was not significant within the BS climate (Figure 4B), and the slopes of the other five continents were heterogeneous. The slopes for Australia and South America were not significantly different from the global estimate of 1.25. The positive correlation between A_mass_ and N_mass_ held across continents within the BS climate (Figure 4C), but the slope varied from 1.42 to 3.22, with only the estimates for Asia and Australia close to the global estimate. In addition, the slopes of the N_mass_–P_mass_ relationships within the BS climate were all relatively high (Figure 4D), except for South America where the fitted slope of 0.61 was insignificantly lower than the global estimate of 0.66.

#### Intercontinental Differences of Leaf Trait Relationship Within the Temperate Humid Climate

**FIGURE 5:**
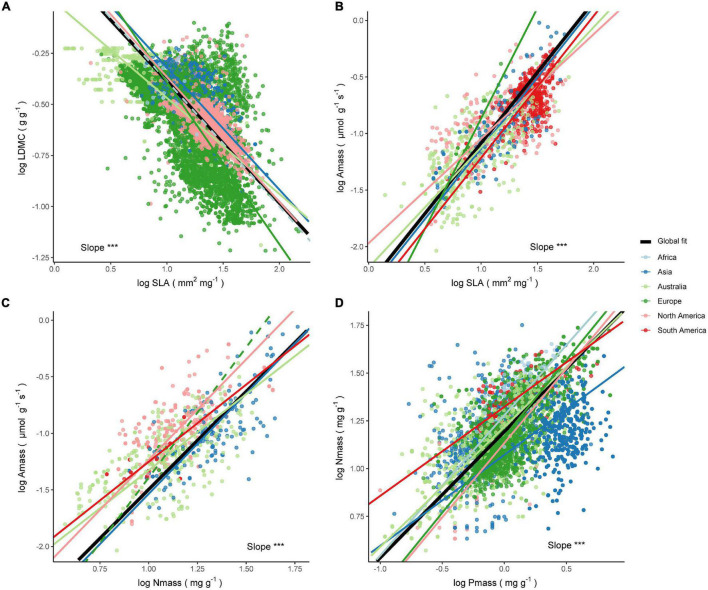
Relationships between leaf economic traits across species within the Cf climate. The significance test results of the slope heterogeneity among the fitted lines are shown (***P < 0.001). The solid line is a significant fit to the standardized major axis (SMA) regression within a continent, while insignificant relationships are shown as dashed lines. Black solid lines are SMA regression lines for all species on a global scale. The respective slopes, sample sizes, and coefficients of determination of the SMA regressions are as follows (all *P* < 0.001 unless noted): **(A)** LDMC–SLA relationship. Africa: *b* = −0.61, *n* = 18, *R*^2^ = 0.01, *P* = 0.67; Asia: *b* = −0.6, *n* = 1304, *R*^2^ = 0.37; Australia: *b* = −0.47, *n* = 533, *R*^2^ = 0.49; Europe: *b* = −0.79, *n* = 9796, *R*^2^ = 0.33; North America: *b* = −0.61, *n* = 2564, *R*^2^ = 0.48. **(B)** A_mass_–SLA relationship. Asia: *b* = 1.25, *n* = 155, *R*^2^ = 0.63; Australia: *b* = 1.06, *n* = 331, *R*^2^ = 0.37; Europe: *b* = 1.95, *n* = 24, *R*^2^ = 0.87; North America: *b* = 0.92, *n* = 307, *R*^2^ = 0.34; South America: *b* = 1.25, *n* = 516, *R*^2^ = 0.46. **(C)** A_mass_–N_mass_ relationship. Asia: *b* = 1.77, *n* = 149, *R*^2^ = 0.57; Australia: *b* = 1.35, *n* = 326, *R*^2^ = 0.4; Europe: *b* = 2.3, *n* = 13, *R*^2^ = 0.06; North America: *b* = 1.78, *n* = 310, *R*^2^ = 0.4; South America: *b* = 1.37, *n* = 21, *R*^2^ = 0.31, *P* = 0.01. **(D)** N_mass_–P_mass_ relationship. Africa: *b* = 0.73, *n* = 219, *R*^2^ = 0.44; Asia: *b* = 0.48, *n* = 1670, *R*^2^ = 0.07; Australia: *b* = 0.63, *n* = 851, *R*^2^ = 0.4; Europe: *b* = 0.8, *n* = 1825, *R*^2^ = 0.46; North America: *b* = 0.78, *n* = 91, *R*^2^ = 0.47; South America: *b* = 0.47, *n* = 103, *R*^2^ = 0.45.

The four trait relationships did not share a common slope across continents within the Cf climate (all *P* < 0.001), and comparisons between continents tend to exhibit slope heterogeneity (Supplementary Table 7). The LDMC–SLA relationship within the Cf climate decoupled in Africa, while the slope shifted from −0.47 to −0.79 within other continents (Figure 5A). There was no significant shift in the slope in Asia and North America compared to the global estimate of 0.6 (Supplementary Table 8). It was interesting to note that for the A_mass_-SLA relationship, the slope estimates for both Asia and South America were highly consistent with the global estimate of 1.25 (Figure 5B). The A_mass_–N_mass_ relationship showed less variation, exhibiting a slope close to the global one of 1.73 (Figure 5C), except for Australia where the slope estimate of 1.35 was significantly smaller than the other continental estimates and the global estimate. Furthermore, the slope of the N_mass_–P_mass_ relationship for Australia was close to the global estimate of 0.66 (Figure 5D), but other continents did not show a converging trend as the slope changes from 0.47 to 0.8 (Figure 5D).

## Discussion

### Variation in Leaf Trait and Shifts in Trait Relationships

Over a long period of evolution and development, plants have developed many functionally relevant intrinsic and extrinsic traits that reflect their ability to adapt to their environment (Cornelissen et al., 2003). This study shows that the range of variation in different leaf economic traits is different (Table 2). For instance, as a pair of closely related structural traits, LDMC has much less variation than SLA, which is consistent with previous reports (Poorter et al., 2010; Perez-Harguindeguy et al., 2013). Indeed, variation in traits can arise from three sources: phenotypic plasticity, intraspecific variation, and interspecific variation (Weiner, 2004; Auger and Shipley, 2013). This suggests that phenotypic plasticity and intraspecific variation are adequately accounted for when using different individual trait values for each species within a site, whereas using the mean of traits for all species within a site represents differences in trait variation across sites mainly in species composition. As our study shows, individual species trait values have larger coefficients of variation (CV) than site mean trait values, and SMA regressions between the former are more indicative of trade-off strategies across species (Wright et al., 2005b).

The LES quantified the classical trade-off theory in ecology based on scaling relationships between functional traits, which allowed us to understand the adaptive strategies of plants between rapid growth and resource conservation in terms of leaf tissue investment and carbon gain (Wright et al., 2004). Our findings supported this global result with a larger sample size analysis, but reveal a clear shift in trait relationships across different climate regions worldwide. Indeed, this is not the first time that global leaf trait relationships have been observed to be shifted at smaller subgroups. Wright et al. (2001) found that soil nutrient and rainfall conditions led to shifts in leaf trait relationships and highlighted that species in dry habitats have water conservation strategies that differ from those in wetter areas. Also, Wright et al. (2005b) found a notable climate-related trend in the A_mass_–N_mass_ relationship, with the SMA slope becoming flatter with increasing mean annual temperature, potential evapotranspiration, or irradiance, with shifts in the log-log slope between about 1.4 and 2. Heberling and Fridley (2012) compared the leaf trait relationships of several different floristic regions around the world and found that A_mass_–N_mass_ relationships showed significant differences, with Northern Hemisphere showing significantly higher SMA slopes than Southern Hemisphere, and Eastern North American showing SMA slopes homogeneous with East Asian, but with significantly higher intercepts. Our study confirms that the highly correlated bivariate relationships for leaf traits differ across climate regions and even within continents, with SMA slopes fluctuating up and down the slope of the global estimates. The leaf economic trait relationship is a reflection of the optimization of plant resource use under a range of environmental conditions (Heberling and Fridley, 2012). Differences in resource availability may cause plants to adjust their traits and the functional focus of the traits, which is a shift in plant adaptation strategies. In the recently developed study of plant trait networks, the shift in the relationships between traits has been given a new way of understanding (Flores-Moreno et al., 2019; He et al., 2020). For example, in cold-temperate forests, the network formed between multiple traits is loose, whereas in tropical forests the plant trait network is tight and complex (He et al., 2020). In this sense, plants in different climatic zones have reason to use flexible or different strategies to cope with their local environment.

### Climate Modulation of Leaf Trait Relationships

The influence of climatic factors on leaf traits has long been of interest to ecologists. Evergreen species in arid areas usually have hardened leaves (Maksimov, 1929; Knight, 1932). In order to conserve water and nutrients, plants usually exhibit a low SLA with a long leaf life span, low A_mass_, and slow growth rate (Reich et al., 1992; Poorter et al., 2009; de la Riva et al., 2016). We found that this was indeed the case in the Arid climate zone and the Polar climate zone (and the species had a higher LDMC), but that SLA was not necessarily be smaller for species with water deficits (Supplementary Table 2). It may be that species within climate types with seasonal water deficits have diverse adaptive strategies (Hoffmann et al., 2005). Specifically, the variation in leaf economic traits is poorly explained in deciduous shrub and tree species (Wright et al., 2004), and these species may be able to maintain lower leaf construction costs (higher SLA) and higher potential payback capacity (higher Amass) to cope with the unfortunate dry season (Franco et al., 2005). Wright et al. (2005b) saw a positive correlation between SLA and mean annual temperature. There is a similar trend in our comparisons between climate zones, with the exception of the Polar climate. However, these researchers graded individual climate factors, which was not a good predictor of trends in bivariate relationships in a given region because the relationship between SMA slope and climate becomes confounded under the compounding influence of various factors. The method of classifying climate regions used in our study is perhaps more comprehensive and representative. Moreover, Reich and Oleksyn (2004) found a pattern that leaf nitrogen content and leaf phosphorus content decreased with increasing nearness to the equator and average temperature. We found that species within the Polar climate had higher N_mass_ and lower P_mass_, as well as species in the Temperate climate had lower N_mass_ and higher P_mass_, which may be difficult to explain by a single average temperature factor.

In this study, we observed shifts in the slope of all leaf economic trait relationships across different climate regions, suggesting that differences in precipitation and temperature do modulate the resource capture strategies of plants and that previous studies may have underestimated the effect of climate on leaf economic trait relationships (Reich et al., 1997; Wright et al., 2004). For instance, although species in both the Tropical climate and the Polar climate exhibited lower SLA and higher LDMC, the LDMC–SLA relationships in these two climate zones were very different. For a given global average SLA (20 mm^2^ mg^–1^), the LDMC within the Polar climate was c. 4.6-fold greater than the LDMC in the Tropical climate (Figure 3A). LDMC within the Ds climate type (lower temperature and summer water deficit) was the highest for a given SLA. This differentiated investment in leaf structural traits may imply that plants in cold environments require greater stress tolerance and resistance to hazards (Cornelissen et al., 2003; Kleyer et al., 2008). In terms of the A_mass_–SLA relationships, differences in moisture have important implications (Figure 3B). For example, the Tropical Monsoon climate (Am) and the Desert climate (BW) had significantly different slopes from most climate types (*b* = 0.83; *b* = 1.54, respectively) and were the two extremes of deviation from the global estimate of 1.25. This suggests that species in extreme arid regions can take full advantage of the limited leaf construction cost to obtain a greater carbon return, often accompanied by higher N_mass_ and leaf-level water use efficiency (Brouillette et al., 2014). There are many possible reasons why species exhibit low photosynthetic capacity within the Tropical Monsoon climate, such as they may invest more in hydraulic transport capacity to cope with the large variability in water availability, and they may invest more in the mechanical organization to increase leaf longevity and tolerate stress and herbivore threats (Warren and Adams, 2004). The A_mass_–N_mass_ relationships seemed to show a climate-related tend, with an increasingly steep slope from the Tropical climate to the Polar climate. This may indicate that the leaf nitrogen allocated to the photosynthetic apparatus is significantly different among species in different climate regions (Evans, 1989; Hikosaka et al., 1998), and this variation can be reflected by differences in the photosynthetic nitrogen-use efficiency. Similar results were found in the global-scale study by Ali et al. (2015), which showed that species from tropical zones tend to have low photosynthetic capacity while species from higher latitudes have high photosynthetic capacity. Furthermore, the relationship between N_mass_ and P_mass_ did not show a monotonic shift trend across either climate zones or climate types. Unexpectedly, the SMA slopes were higher than the global estimate of 0.66 in many climate zones, which is significantly different from the metabolic theory prediction of 2/3 (Reich et al., 2010). Our results suggest that bivariate leaf trait relationships across climate regions are sometimes inconsistent with broader patterns, indicating that global analyses need to be applied with caution to regions or localities with specific environmental conditions (Wigley et al., 2016). Moreover, the results of such subgroup analysis are quite important because it reminds us to avoid deriving the patterns of the world LES under Simpson’s paradox (Simpson, 1951).

### Biogeographic Constraints on Leaf Trait Relationships

Our second scientific question concerns the biogeographic constraints of leaf trait relationships. It has been found that different leaf trait relationships exist in some flora with different evolutionary histories (Heberling and Fridley, 2012). Despite the fact that continents are not a strict flora boundary, the species composition of different continents has varied considerably over a long evolutionary history, so that cross-species leaf economic trait relationships may exhibit diverse patterns under different selection pressures (Donovan et al., 2011). We found that the A_mass_–N_mass_ relationships within different continents were relatively close to global estimates, but that other leaf trait relationships diverged relatively widely across different continents (Supplementary Table 8). Within the BS climate, leaf trait relationships were a contrasting set in Africa and North America. For a given SLA (global mean of 20 mm^2^ mg^–1^), the LDMC of species in Africa was 1.5 times higher and the A_mass_ was 1.9 times higher compared to species in North America, in addition to a higher scaling exponent between N_mass_ and P_mass_ (0.97 vs. 0.94). Within the Cf climate, the contrast between all leaf trait relationships in Australia and Europe was remarkable. Species in Europe had a similar LDMC but 3.3 times higher A_mass_ at a given SLA of 20 mm^2^ mg^–1^ compared to species in Australia. For a given N_mass_ (global mean of 20 mg g^–1^), the A_mass_ of species in Europe was 1.7 times higher than that of species in Australia, as well as a higher scaling exponent of N_mass_–P_mass_ relationship (0.8 vs. 0.63).

In our study, differences in species composition may contribute to the intercontinental differences in SMA slope (Supplementary Figure 3). For instance, herbaceous species were the most sampled in Asia and North America within the BS climate, while evergreen broad-leaved woody species dominated in Europe; within the Cf climate, the samples from South America were almost evergreen broadleaf species, while North America was dominated by deciduous broadleaf species. Previous studies have shown that species of different functional groups can be very different in their strategies. For example, evergreen woody species are often more slow-growing and resource-conservative, while herbaceous species tend to be more fast-growing and resource-acquisitive (Poorter et al., 2009; Ghimire et al., 2017). In addition, differences in soil properties may also be important factors influencing the bivariate relationships of leaf traits (Fonseca et al., 2000; Wright et al., 2001; Ordoñez et al., 2009). Although climate is an important driver of soil development (Jenny, 1994), and soil pH, total nitrogen, and total phosphorus vary predictably across precipitation gradients (Huston, 2012), soil properties have independent contributions to leaf traits. This may also be one of the reasons for the appearance of intercontinental differences. Patterns of terrestrial nitrogen and phosphorus limitation help explain this view, for example, within the BS climate type, sites in Australia exhibited mainly phosphorus limitation, while sites in Asia exhibit predominantly nitrogen limitation (Du et al., 2020). Furthermore, Maire et al. (2015) found that leaf nitrogen content and leaf phosphorus content (both on the area basis) were more affected by the joint effects of soil and climate and that the independent effect of soil on maximum leaf photosynthetic rate on the area basis (A_*area*_) was greater than the independent effect of climate, indicating the importance of quantifying the effect of soil factors on leaf economic traits. Several studies have shown that sample size effects and differences in light intensity may also affect leaf trait relationships (Cunningham et al., 1999; Wright et al., 2005a; Keenan and Niinemets, 2016), and we have not yet fully considered the impact of these factors on intercontinental comparisons, which need to be refined in future studies and linked to homogeneous garden experiments to further explain regional differences in leaf trait relationships (Agrawal et al., 2015). Finally, we expect these results to provide contribution to the calibration and scale transformation of earth system models.

## Data Availability Statement

The original contributions presented in the study are included in the article/Supplementary Material, further inquiries can be directed to the corresponding author.

## Author Contributions

YH and LR conceived the study. LR performed the analyses. All authors contributed to the interpretation of the results and to the text.

## Conflict of Interest

The authors declare that the research was conducted in the absence of any commercial or financial relationships that could be construed as a potential conflict of interest.

## Publisher’s Note

All claims expressed in this article are solely those of the authors and do not necessarily represent those of their affiliated organizations, or those of the publisher, the editors and the reviewers. Any product that may be evaluated in this article, or claim that may be made by its manufacturer, is not guaranteed or endorsed by the publisher.

## Footnotes

1
https://www.try-db.org
