# Supplementary material for: Differential Investment Strategies in Leaf Economic Traits Across Climate Regions Worldwide
Source: Front Plant Sci. 2022 Mar 4;13:798035. doi: 10.3389/fpls.2022.798035 (PMC8959930; doi:10.3389/fpls.2022.798035)
Supplement: Supplementary file 1 [file Data_Sheet_1.docx]

Supplementary Material

# Supplementary Figures


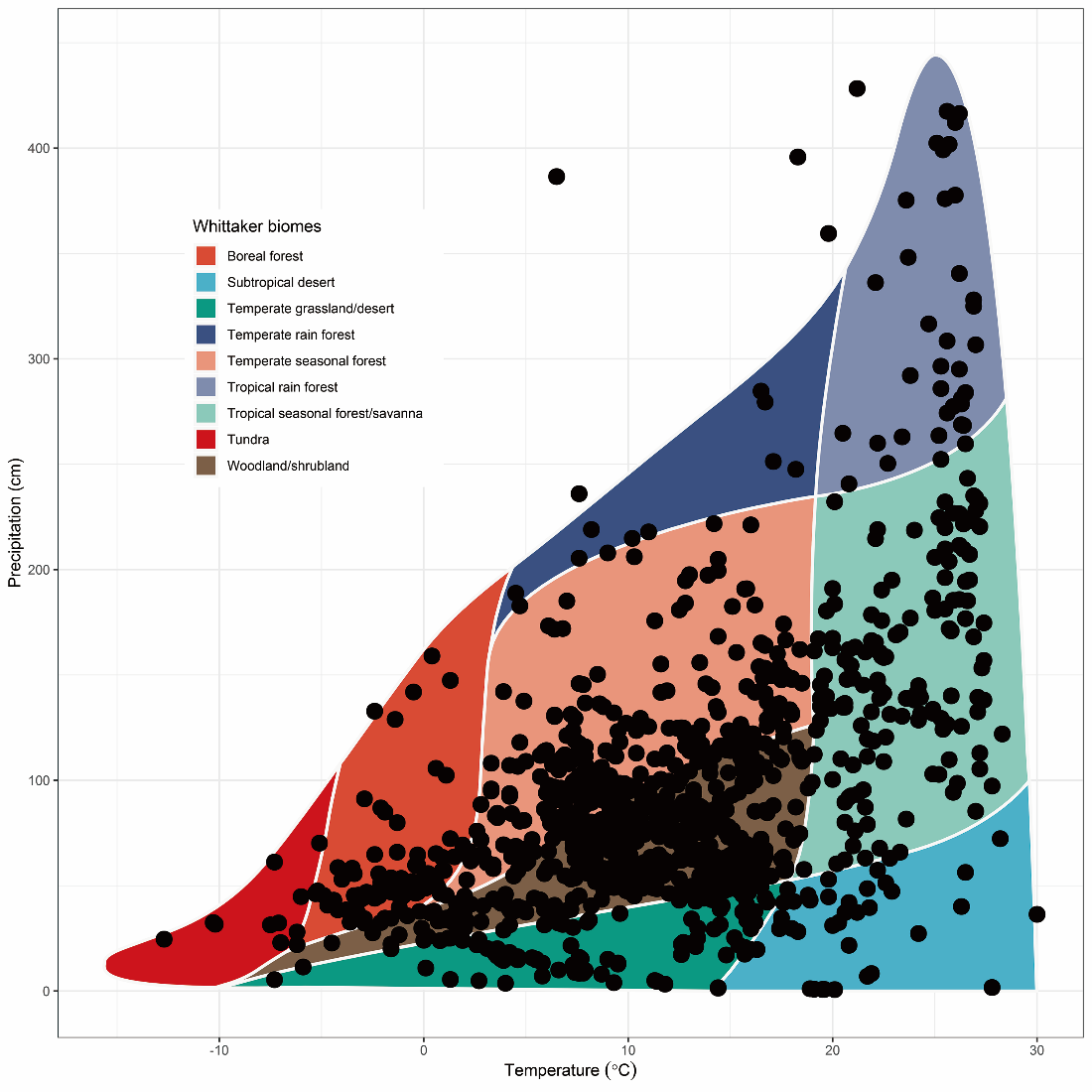


**Supplementary Figure 1.** The climatic space occupied by all observations in terms of mean annual temperature and mean annual precipitation and their corresponding biomes.


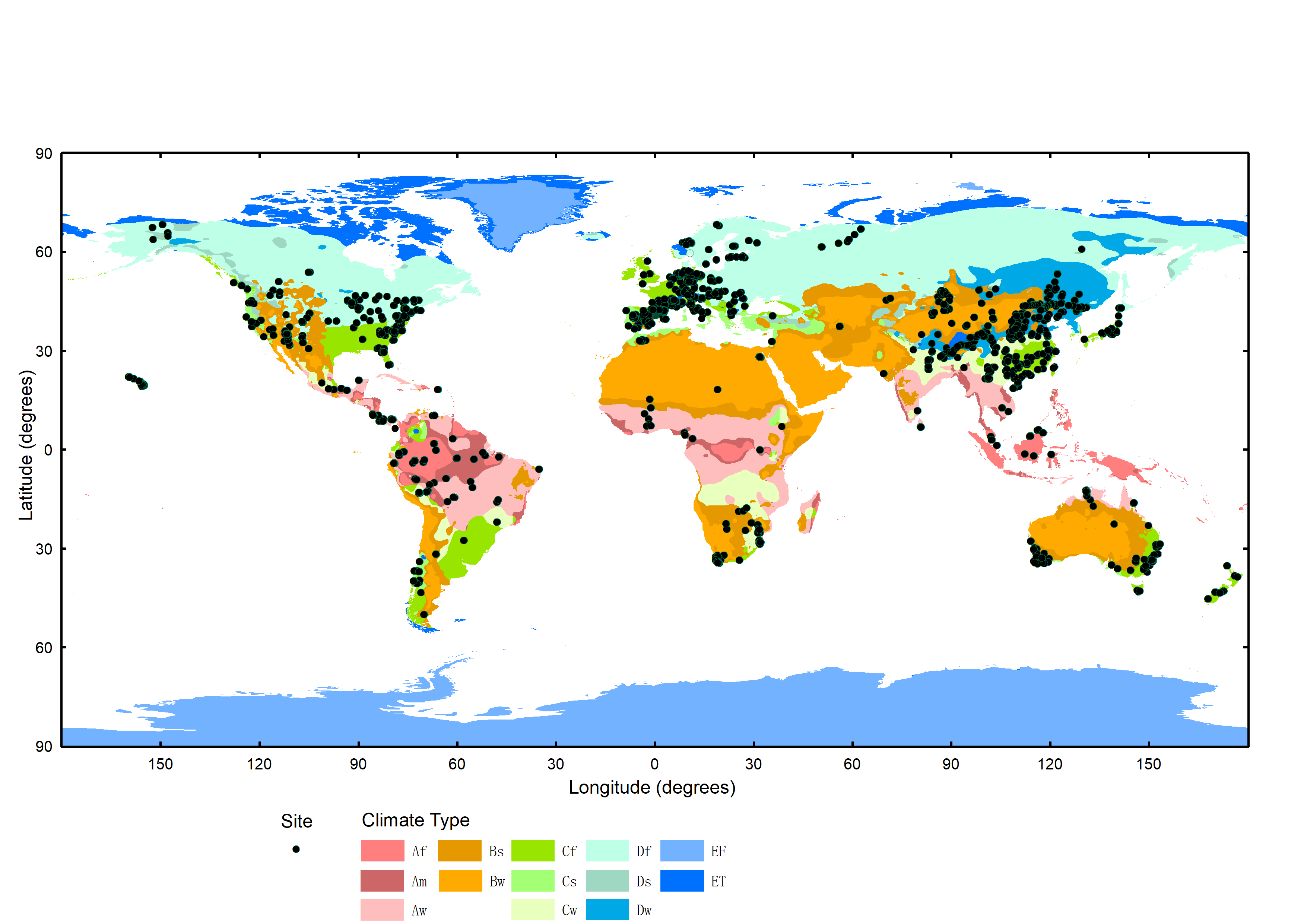


**Supplementary Figure 2.** Distribution of the sites to which the leaf traits belong in different climate types of the world.

**
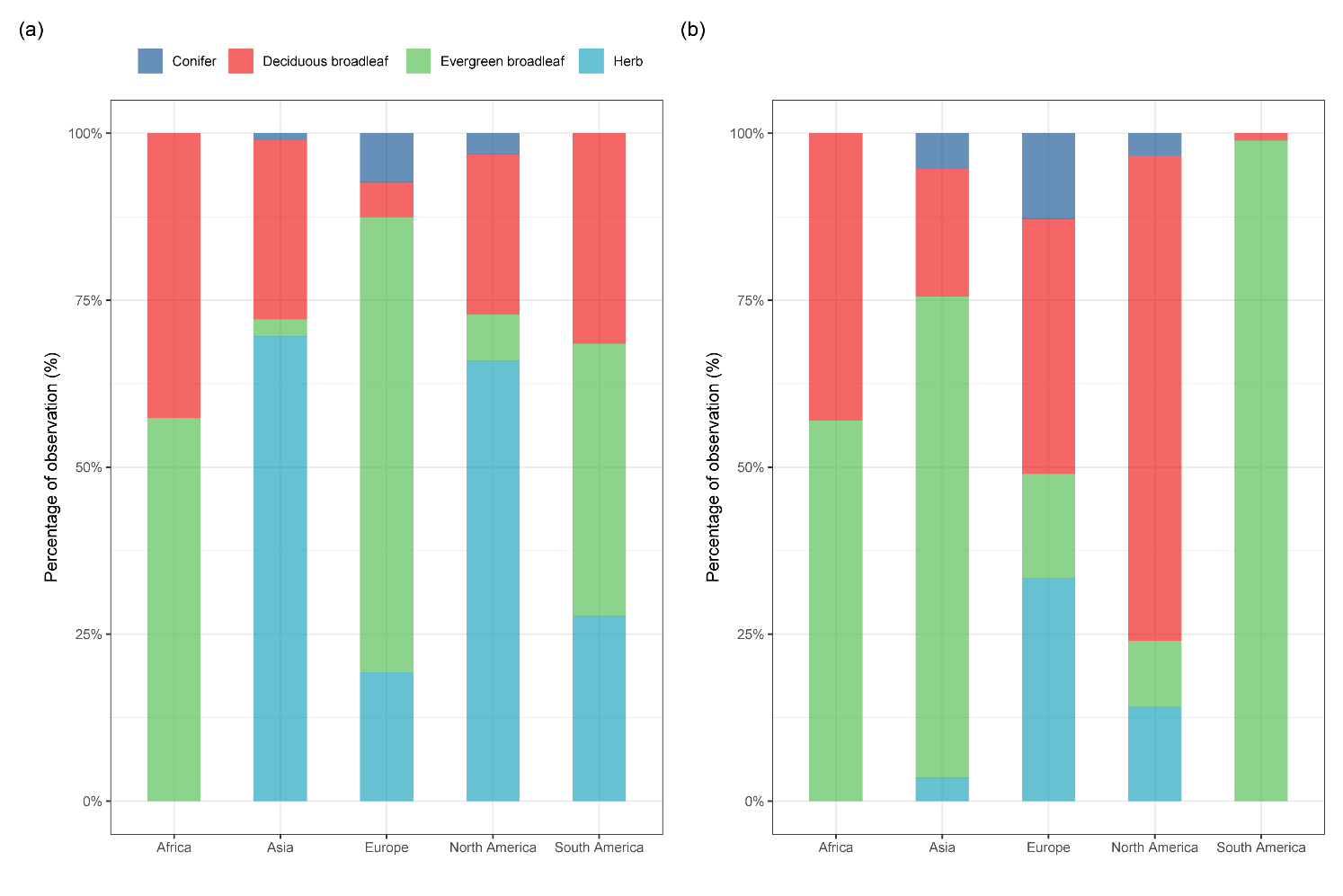
**

**Supplementary Figure 3.** Differences in species composition between the six continents within the BS climate type (a) and the Cf climate type (b). These four functional groups (conifers, evergreen broadleaf woody species, deciduous broadleaf woody species and herb) represent the vast majority of the samples within the dataset, whereas some species with relatively small percentages or unknown taxa are ignored here.

# Supplementary Tables

**Supplementary Table 1.** Compiled list of datasets, including the original publication and dataset ID in TRY (https://www.try-db.org/).

| **Dataset ID** | **Dataset** | **Publication reference** |
| --- | --- | --- |
| 20 | GLOPNET - Global Plant Trait Network Database | Wright *et al.*, 2004 |
| 37 | Sheffield Database | Cornelissen *et al.*, 1996 |
| 47 | South African Woody Plants Database (ZLTP) | unpublished |
| 50 | Leaf and Whole Plant Traits Database | Shipley, 2002 |
| 51 | Tropical Plant Traits From Borneo Database | Swaine & E., 2007 |
| 67 | Leaf Physiology Database | Kattge *et al.*, 2009 |
| 80 | French Massif Central Grassland Trait Database | Louault *et al.*, 2005 |
| 88 | The Netherlands Plant Traits Database | Ordonez *et al.*, 2010 |
| 105 | Traits from Subarctic Plant Species Database | Freschet *et al.*, 2010 |
| 112 | Panama Plant Traits Database | Wright *et al.*, 2010 |
| 113 | Panama Leaf Traits Database | Messier *et al.*, 2010 |
| 130 | Global 15N Database | Craine *et al.*, 2009 |
| 150 | French Weeds Trait Database | unpublished |
| 152 | Functional Traits of Graminoids in Semi-Arid Steppes Database | Adler *et al.*, 2004 |
| 159 | Traits of US Desert Woody Plant Species | Butterfield & Briggs, 2011 |
| 161 | Leaf Traits in Central Apennines Beech Forests | Campetella *et al.*, 2011 |
| 180 | Leaf Ash Content in China's Terrestrial Plants | Han *et al.*, 2012 |
| 181 | Leaf Nitrogen and Phosphorus for China's Terrestrial Plants | Chen *et al.*, 2013 |
| 193 | Plant Traits for Pinus and Juniperus Forests in Arizona | Laughlin *et al.*, 2011 |
| 223 | San Lorenzo Epiphyte Leaf Traits Database | Petter *et al.*, 2016 |
| 230 | Panama Tree Traits | Craven *et al.*, 2007 |
| 234 | Leaf Traits Mount Hutt, New Zealand | Kichenin *et al.*, 2013 |
| 236 | Chinese Traits | Prentice *et al.*, 2011 |
| 237 | Harze Trait Intravar: SLA, LDMC and Plant Height for Calcareous Grassland Species in South Belgium | unpublished |
| 240 | Nutrient Resorption Efficiency Database | Vergutz *et al.*, 2012 |
| 252 | Leaf Structure and Chemistry | Auger & Shipley, 2013 |
| 263 | Costa Rican Tropical Dry Forest Trees | Powers & Tiffin, 2010 |
| 266 | Hawaiian Lobeliad | Montgomery & Givnish, 2008 |
| 272 | Plant Coastal Dune Traits (France, Aquitaine) | unpublished |
| 275 | Plant Traits From Spanish Mediteranean shrublands | unpublished |
| 288 | CTFS Luquillo Forest Dynamics Plot | Swenson *et al.*, 2011 |
| 291 | MARGINS - leaf traits database | unpublished |
| 293 | Jasper Ridge leaf chemistry data | Dahlin *et al.*, 2013 |
| 295 | Leaf functional traits in the Hawaiian silversword alliance | Blonder *et al.*, 2016 |
| 296 | Northern mixed-grass prairie species traits - Wyoming, USA | unpublished |
| 297 | Traits of Polygonum viviparum L. | Boucher *et al.*, 2013 |
| 309 | Plant traits of grassland species | La Pierre & Smith, 2015 |
| 310 | French Alps Trait Data | Gos *et al.*, 2016 |
| 311 | Structural and biochemical leaf traits of boreal tree species in Finland | Lukes *et al.*, 2013 |
| 315 | Leaf traits for Picea glauca and Pinus sylvestris on University of Calgary (Canada) campus | Michaletz & Johnson, 2006 |
| 320 | Grassland Plant Trait Database | Takkis, 2014 |
| 323 | Rocky Mountain Biological Laboratory WSR/gradient plant traits | unpublished |
| 326 | Leaf nutrient concentrations | Rolo *et al.*, 2012 |
| 328 | Root Traits of Grassland Species | Smith *et al.*, 2014 |
| 331 | Traits of savannah trees in the Hluhluwe-iMfolozi Game reserve, South Africa | van der Plas & Olff, 2014 |
| 332 | Photosynthetic parameters, respiration and leaf traits of a Peruvian tropical montane cloud forest | van de Weg *et al.*, 2012 |
| 333 | LMA, leaf tissue density and N&P content along the Amazon-Andes gradient in Peru | van de Weg *et al.*, 2009 |
| 334 | A Global Data Set of Leaf Photosynthetic Rates, Leaf N and P, and Specific Leaf Area | Walker *et al.*, 2014 |
| 342 | Photosynthesis Traits Worldwide | Maire *et al.*, 2015 |
| 344 | LosTuxtlas_functionaltraits | Martinez-Garza *et al.*, 2013 |
| 351 | Miombo tree species - SLA, leaf and seed size | Joseph *et al.*, 2014 |
| 352 | Miombo tree species - leaf nutrients | Seymour *et al.*, 2014 |
| 353 | Old fields of Eastern US (Siefert Data) | Siefert *et al.*, 2014 |
| 367 | Tree species functional traits from Dinghushan Biosphere Reserve, southern China | Li *et al.*, 2015 |
| 374 | Traits of fertile (spore bearing) leaves of rainforest ferns from El Verde Field, Puerto Rico | Sharpe & N., 2016 |
| 375 | Traits of sterile (non-spore bearing) leaves of rainforest ferns from El Verde Field, Puerto Rico | Chacon-Madrigal *et al.*, 2018 |
| 377 | Functional Traits of Trees in Golfo Dulce, Costa Rica | Chacon-Madrigal *et al.*, 2018 |
| 378 | Traits of the Hungarian flora | Lhotsky *et al.*, 2016 |
| 403 | Iranian Plant Trait Dataset | unpublished |
| 412 | The Global Leaf Traits | unpublished |
| 415 | Ozark glade grassland plants | Miller *et al.*, 2018 |
| 419 | Sherbrooke | Li & Shipley, 2018 |
| 422 | Hawaii native and non indigenous species. Traits and environment | Baruch & Goldstein, 1999 |
| 427 | Coffea arabica var. Caturra - leaf traits | Buchanan *et al.*, 2019 |
| 428 | Trait and biomass data 2014 and 2015 of the BE_LOW project | Herz *et al.*, 2017 |
| 443 | The China Plant Trait Database | Wang *et al.*, 2017 |
| 447 | Herbaceous plants of Rouge National Urban Park | unpublished |
| 449 | KIT herbaceous functional gradient (median) | Kattenborn *et al.*, 2019 |
| 453 | European North Russia | Dalke *et al.*, 2018 |
| 454 | Leaf traits from ECOSHRUB Dovrefjell Norway | unpublished |

**Supplementary Table 2.** Summary of leaf economic traits across different climate regions based on individual mean values.

| **Climate regions** | | **SLA**  **(mm^2^ mg^-1^)** | | | **LDMC**  **(g g^-1^)** | | | **A_mass_**  **(μmol g^-1^s-1)** | | | **N_mass_**  **(mg g^-1^)** | | | **P_mass_**  **(mg g^-1^)** | | |
| --- | --- | --- | --- | --- | --- | --- | --- | --- | --- | --- | --- | --- | --- | --- | --- | --- |
|  |  | Mean | SD | CV (%) | Mean | SD | CV (%) | Mean | SD | CV (%) | Mean | SD | CV (%) | Mean | SD | CV (%) |
| **A** |  | 16.1 | 7.55 | 46.9 | 0.34 | 0.1 | 29.4 | 0.12 | 0.11 | 91.7 | 20.05 | 7.93 | 39.6 | 0.98 | 0.56 | 57.1 |
|  | Af | 15.18 | 6.63 | 43.7 | 0.32 | 0.09 | 28.1 | 0.11 | 0.1 | 90.9 | 21.06 | 8.76 | 41.6 | 1 | 0.57 | 57.0 |
|  | Am | 16.62 | 7.76 | 46.7 | 0.33 | 0.1 | 30.3 | 0.1 | 0.06 | 60.0 | 19.4 | 7.51 | 38.7 | 0.99 | 0.58 | 58.6 |
|  | Aw | 12.83 | 6.44 | 50.2 | 0.39 | 0.09 | 23.1 | 0.16 | 0.15 | 93.8 | 20.11 | 7.37 | 36.6 | 0.96 | 0.52 | 54.2 |
| **B** |  | 11.3 | 10.67 | 94.4 | 0.32 | 0.12 | 37.5 | 0.12 | 0.11 | 91.7 | 21.65 | 8.71 | 40.2 | 1.5 | 0.77 | 51.3 |
|  | BW | 13.24 | 14.31 | 108.1 | 0.29 | 0.11 | 37.9 | 0.22 | 0.15 | 68.2 | 21.8 | 10.45 | 47.9 | 1.3 | 0.67 | 51.5 |
|  | BS | 9.97 | 6.88 | 69.0 | 0.35 | 0.13 | 37.1 | 0.12 | 0.1 | 83.3 | 21.61 | 8.31 | 38.5 | 1.53 | 0.79 | 51.6 |
| **C** |  | 19.38 | 12.05 | 62.2 | 0.3 | 0.11 | 36.7 | 0.13 | 0.1 | 76.9 | 17.15 | 7.46 | 43.5 | 1.56 | 1.17 | 75.0 |
|  | Cs | 24.91 | 15.5 | 62.2 | 0.27 | 0.11 | 40.7 | 0.09 | 0.08 | 88.9 | 16.51 | 7.61 | 46.1 | 1.24 | 0.97 | 78.2 |
|  | Cw | 14.19 | 8.41 | 59.3 | 0.35 | 0.1 | 28.6 | 0.14 | 0.1 | 71.4 | 19.06 | 7.44 | 39.0 | 1.79 | 1.23 | 68.7 |
|  | Cf | 17.98 | 10.27 | 57.1 | 0.32 | 0.1 | 31.3 | 0.15 | 0.11 | 73.3 | 16.76 | 7.34 | 43.8 | 1.54 | 1.17 | 76.0 |
| **D** |  | 25.79 | 13.43 | 52.1 | 0.3 | 0.09 | 30.0 | 0.18 | 0.11 | 61.1 | 22.64 | 8.92 | 39.4 | 1.85 | 1.06 | 57.3 |
|  | Ds | 9.39 | 7.43 | 79.1 | 0.42 | 0.11 | 26.2 | 0.07 | 0.04 | 57.1 | 20.64 | 9.38 | 45.4 | 1.95 | 1.29 | 66.2 |
|  | Dw | 26.6 | 14.61 | 54.9 | 0.29 | 0.1 | 34.5 | 0.18 | 0.1 | 55.6 | 23.41 | 8.22 | 35.1 | 1.91 | 1.04 | 54.5 |
|  | Df | 26.09 | 13.24 | 50.7 | 0.3 | 0.09 | 30.0 | 0.18 | 0.11 | 61.1 | 22.61 | 9.09 | 40.2 | 1.76 | 0.98 | 55.7 |
| **E** | ET | 15.85 | 7.91 | 49.9 | 0.37 | 0.12 | 32.4 | 0.19 | 0.12 | 63.2 | 23.13 | 9.64 | 41.7 | 1.27 | 0.72 | 56.7 |

**Supplementary Table 3.** Fitting of SMA slopes for different climate zones based on site mean trait values and comparison with global estimates. A '↑' indicates the slope is significantly higher than the global estimate (P < 0.05), '↓' indicates the slope is significantly lower than the global estimate (P < 0.05), '−' indicates the slope is not significantly different from the global estimate (P > 0.05).

| **Climate zones** | **Slope (95% CIs)** | **N** | **R^2^** | **P** | **Compared to all** |
| --- | --- | --- | --- | --- | --- |
| **(a) LDMC–SLA relationships** | | | | | |
| A | - 0.52 (- 0.67, - 0.40) | 31 | 0.51 | < 0.001 | − |
| B | - 0.57 (- 0.69, - 0.47) | 110 | 0.01 | 0.42 |  |
| C | - 0.70 (- 0.81, - 0.61) | 112 | 0.44 | < 0.001 | ↓ |
| D | - 0.62 (- 0.70, - 0.54) | 124 | 0.43 | < 0.001 | − |
| E | - 0.41 (- 0.63, - 0.27) | 4 | 0.98 | < 0.05 | − |
| **(b) A_mass_–SLA relationships** | | | | | |
| A | 1.23 (0.87, 1.76) | 26 | 0.27 | < 0.01 | − |
| B | 2.01 (1.00, 4.06) | 7 | 0.56 | 0.05 |  |
| C | 1.11 (0.89, 1.38) | 41 | 0.54 | < 0.001 | − |
| D | 1.41 (1.02, 1.96) | 36 | 0.08 | 0.09 |  |
| E | 0.93 (0.44, 1.97) | 3 | 0.99 | < 0.05 | − |
| **(c) A_mass_–N_mass_ relationships** | | | | | |
| A | 1.59 (1.13, 2.24) | 20 | 0.51 | < 0.001 | − |
| B | 2.14 (1.53, 3.00) | 9 | 0.83 | < 0.001 | − |
| C | 1.54 (1.24, 1.90) | 32 | 0.66 | < 0.001 | − |
| D | 2.26 (1.67, 3.06) | 29 | 0.40 | < 0.001 | − |
| E | 2.21 (1.17, 4.17) | 6 | 0.76 | < 0.05 | − |
| **(d) N_mass_–P_mass_ relationships** | | | | | |
| A | 0.70 (0.57, 0.85) | 77 | 0.29 | < 0.001 | − |
| B | 0.79 (0.69, 0.91) | 73 | 0.67 | < 0.001 | ↑ |
| C | 0.71 (0.64, 0.78) | 134 | 0.69 | < 0.001 | − |
| D | 0.92 (0.78, 1.09) | 63 | 0.58 | < 0.001 | ↑ |
| E |  | 1 |  |  |  |

**Supplementary Table 4.** Multiple comparisons between SMA slopes for different climate zones based on individual trait values. Different lowercase letters indicate a significant difference at P < 0.05.

| **Climate zones** | **Multiple comparisons between subgroups** | **P-value (Compared to all)** |
| --- | --- | --- |
| **(a) LDMC–SLA relationships** | | |
| A | c | < 0.001 |
| B | a | ns |
| C | b | < 0.001 |
| D | a | < 0.001 |
| E | a | ns |
| **(b) A_mass_–SLA relationships** | | |
| A | ab | < 0.001 |
| B | a | < 0.001 |
| C | c | < 0.001 |
| D | b | < 0.001 |
| E | ab | ns |
| **(c) A_mass_–N_mass_ relationships** | | |
| A | c | < 0.001 |
| B | a | < 0.001 |
| C | b | ns |
| D | a | < 0.001 |
| E | a | < 0.001 |
| **(d) N_mass_–P_mass_ relationships** | | |
| A | b | < 0.001 |
| B | a | < 0.001 |
| C | c | < 0.001 |
| D | b | < 0.001 |
| E | bc |  |

**Supplementary Table 5.** Matrix of pair-wise comparisons between climate types based on individual trait values. A '***' indicates P < 0.001, '**' indicates 0.001 < P < 0.01, '*' indicates 0.01 < P < 0.05, '.' indicates 0.05 < P < 0.1, ' ' indicates P > 0.1.

| **(a) LDMC ~ SLA** | | | | | | | | | | | | |
| --- | --- | --- | --- | --- | --- | --- | --- | --- | --- | --- | --- | --- |
|  | Af | Am | Aw | BS | BW | Cf | Cs | Cw | Df | Ds | Dw | ET |
| Af | - | *** | *** | * | *** |  | . |  | *** | *** |  | *** |
| Am | *** | - | *** | *** | *** | *** | *** | *** | *** | *** | *** | *** |
| Aw | *** | *** | - | *** |  | *** | *** | *** | *** |  | *** | * |
| BS | * | *** | *** | - | *** | ** |  |  | ** | *** |  |  |
| BW | *** | *** |  | *** | - | *** | *** | *** | *** | *** | *** | . |
| Cf |  | *** | *** | ** | *** | - | ** |  | *** | *** |  | *** |
| Cs | . | *** | *** |  | *** | ** | - |  | *** | *** |  |  |
| Cw |  | *** | *** |  | *** |  |  | - |  | *** |  |  |
| Df | *** | *** | *** | ** | *** | *** | *** |  | - | *** | *** |  |
| Ds | *** | *** |  | *** | *** | *** | *** | *** | *** | - | *** | *** |
| Dw |  | *** | *** |  | *** |  |  |  | *** | *** | - | ** |
| ET | *** | *** | * |  | . | *** |  |  |  | *** |  | - |
| **(b) A_mass_ ~ SLA** | | | | | | | | | | | | |
|  | Af | Am | Aw | BS | BW | Cf | Cs | Cw | Df | Ds | Dw | ET |
| Af | - | *** |  |  |  | *** |  | *** | ** |  |  |  |
| Am | *** | - | *** | *** | *** | . |  | * | *** |  | *** | *** |
| Aw |  | *** | - |  |  | *** |  | ** |  |  |  |  |
| BS |  | *** |  | - |  | *** |  | *** | * |  |  |  |
| BW |  | ** |  |  | - | . |  |  |  |  |  |  |
| Cf | *** | . | *** | *** | . | - |  |  | *** |  | *** | * |
| Cs |  | *** |  |  |  | *** | - | *** |  |  |  |  |
| Cw | *** | * | ** | *** |  |  |  | - | ** |  | * |  |
| Df | ** | *** |  | * |  | *** |  | ** | - |  |  |  |
| Ds |  |  |  |  |  |  |  |  |  | - |  |  |
| Dw |  | *** |  |  |  | *** |  | * |  |  | - |  |
| ET |  | *** |  |  |  | * |  |  |  |  |  | - |
| **(c) A_mass_ ~ N_mass_** | | | | | | | | | | | | |
|  | Af | Am | Aw | BS | BW | Cf | Cs | Cw | Df | Ds | Dw | ET |
| Af | - | * | *** | *** |  | ** | *** | *** | *** |  | *** | *** |
| Am | * | - |  |  |  |  | * |  |  |  |  | . |
| Aw | *** |  | - |  |  | ** | * |  |  |  |  |  |
| BS | *** |  |  | - |  | *** |  |  |  |  |  |  |
| BW |  |  |  |  | - |  |  |  |  |  |  |  |
| Cf | ** |  | ** | *** |  | - | *** | *** | *** |  |  | *** |
| Cs | *** | * | * |  |  | *** | - |  |  |  |  |  |
| Cw | *** |  |  |  |  | *** |  | - |  |  |  |  |
| Df | *** |  |  |  |  | *** |  |  | - |  |  |  |
| Ds |  |  |  |  |  |  |  |  |  | - |  |  |
| Dw | *** |  |  |  |  |  |  |  |  |  | - |  |
| ET | *** | . |  |  |  | *** |  |  |  |  |  | - |
| **(d) N_mass_ ~ P_mass_** | | | | | | | | | | | | |
|  | Af | Am | Aw | BS | BW | Cf | Cs | Cw | Df | Ds | Dw | ET |
| Af | - |  |  |  | *** | *** | . | *** |  | *** | *** |  |
| Am |  | - | ** | * | *** | *** |  | ** |  |  |  |  |
| Aw |  | ** | - |  | *** | *** | *** | *** |  | *** | *** |  |
| BS |  | * |  | - | *** | *** | *** | *** |  | *** | *** |  |
| BW | *** | *** | *** | *** | - | *** | *** | *** |  | *** | *** | *** |
| Cf | *** | *** | *** | *** | *** | - | *** |  |  |  | ** |  |
| Cs | . |  | *** | *** | *** | *** | - | * |  |  |  |  |
| Cw | *** | ** | *** | *** | *** |  | * | - |  |  |  |  |
| Df | *** | *** |  |  | ** | *** | *** | *** | - | *** | *** | . |
| Ds | *** |  | *** | *** | *** |  |  |  | *** | - |  |  |
| Dw | ** |  | *** | *** | *** | ** |  |  | *** |  | - |  |
| ET |  |  |  |  | *** |  |  |  | . |  |  | - |

**Supplementary Table 6.** Matrix of pair-wise comparisons between continents within the BS climate type based on individual trait values. A '***' indicates P < 0.001, '**' indicates 0.001 < P < 0.01, '*' indicates 0.01 < P < 0.05, '.' indicates 0.05 < P < 0.1, ' ' indicates P > 0.1.

| **(a) LDMC ~ SLA** | | | | | | |
| --- | --- | --- | --- | --- | --- | --- |
|  | Africa | Asia | Europe | North America | South America |  |
| Africa | – |  |  | *** |  |  |
| Asia |  | – | *** | *** |  |  |
| Europe |  | *** | – | *** |  |  |
| North America | *** | *** | *** | – | * |  |
| South America |  |  |  | * | – |  |
| **(b) A_mass_ ~ SLA** | | | | | | |
|  | Africa | Asia | Australia | Europe | North America | South America |
| Africa | – | . | *** | *** | ** |  |
| Asia | . | – |  |  |  |  |
| Australia | *** |  | – | . | ** |  |
| Europe | *** |  | . | – |  |  |
| North America | ** |  | ** |  | – |  |
| South America |  |  |  |  |  | – |
| **(c) A_mass_ ~ N_mass_** | | | | | | |
|  | Asia | Australia | Europe | North America | South America |  |
| Asia | – |  |  |  | * |  |
| Australia |  | – |  |  | * |  |
| Europe |  |  | – |  | * |  |
| North America |  |  |  | – |  |  |
| South America | * | * | * |  | – |  |
| **(d) N_mass_ ~ P_mass_** | | | | | | |
|  | Africa | Asia | Australia | Europe | North America | South America |
| Africa | – | ** |  |  |  | ** |
| Asia | ** | – | ** |  |  |  |
| Australia |  | ** | – |  |  | ** |
| Europe |  |  |  | – |  |  |
| North America |  |  |  |  | – | . |
| South America | ** |  | ** |  | . | – |

**Supplementary Table 7.** Matrix of pair-wise comparisons between continents within the BS climate type based on individual trait values. A '***' indicates P < 0.001, '**' indicates 0.001 < P < 0.01, '*' indicates 0.01 < P < 0.05, '.' indicates 0.05 < P < 0.1, ' ' indicates P > 0.1.

| **(a) LDMC ~ SLA** | | | | | | |
| --- | --- | --- | --- | --- | --- | --- |
|  | Africa | Asia | Australia | Europe | North America |  |
| Africa | – |  |  |  |  |  |
| Asia |  | – | *** | *** |  |  |
| Australia |  | *** | – | *** | *** |  |
| Europe |  | *** | *** | – | *** |  |
| North America |  |  | *** | *** | – |  |
| **(b) A_mass_ ~ SLA** | | | | | | |
|  | Asia | Australia | Europe | North America | South America |  |
| Asia | – |  | *** | *** |  |  |
| Australia |  | – | *** |  | * |  |
| Europe | *** | *** | – | *** | *** |  |
| North America | *** |  | *** | – | *** |  |
| South America |  | * | *** | *** | – |  |
|  |  |  |  |  |  | – |
| **(c) A_mass_ ~ N_mass_** | | | | | | |
|  | Asia | Australia | Europe | North America | South America |  |
| Asia | – | *** |  |  |  |  |
| Australia | *** | – |  | *** |  |  |
| Europe |  |  | – |  |  |  |
| North America |  | *** |  | – |  |  |
| South America |  |  |  |  | – |  |
| **(d) N_mass_ ~ P_mass_** | | | | | | |
|  | Africa | Asia | Australia | Europe | North America | South America |
| Africa | – | *** | . |  |  | *** |
| Asia | *** | – | *** | *** | *** |  |
| Australia | . | *** | – | *** |  | ** |
| Europe |  | *** | *** | – |  | *** |
| North America |  | *** |  |  | – | *** |
| South America | *** |  | ** | *** | *** | – |

**Supplementary Table 8.** Comparison of SMA slopes among different continents within the same climate type based on individual trait values. The comparisons only show results where traits are significantly correlated (P < 0.05).

| **within the BS climate** | | | **within the Cf climate** | | |
| --- | --- | --- | --- | --- | --- |
| Climate zones | Slope (95% CIs) | P-value (Compared to all) | Climate zones | Slope (95% CIs) | P-value (Compared to all) |
| **(a) LDMC–SLA relationships** | | | | | |
| Africa | - 0.62 (- 0.79, - 0.49) | 0.77 | Africa | - 0.61 (-1.02, - 0.37) |  |
| Asia | - 0.85 (- 0.90, - 0.80) | < 0.001 | Asia | - 0.60 (- 0.62, - 0.57) | 0.90 |
| Europe | - 0.69 (-0.72, - 0.66) | < 0.001 | Australia | - 0.47 (- 0.50, - 0.44) | < 0.001 |
| North America | - 1.04 (- 1.11, - 0.97) | < 0.001 | Europe | - 0.79 (- 0.81, - 0.78) | < 0.001 |
| South America | - 0.70 (- 0.89, - 0.55) |  | North America | - 0.61 (- 0.63, -0.60) | 0.07 |
| **(b) A_mass_–SLA relationships** | | | | | |
| Africa | 2.35 (2.05, 2.69) | < 0.001 | Asia | 1.25 (1.34, 1.38) | 0.96 |
| Asia | 1.43 (1.05, 1.94) |  | Australia | 1.06 (0.97, 1.16) | < 0.001 |
| Australia | 1.16 (1.06, 1.28) | 0.16 | Europe | 1.95 (1.67, 2.28) | < 0.001 |
| Europe | 1.45 (1.30, 1.63) | < 0.01 | North America | 0.92 (0.84, 1.01) | < 0.001 |
| North America | 1.62 (1.42, 1.84) | < 0.001 | South America | 1.25 (1.18, 1.34) | 0.90 |
| South America | 1.62 (1.23, 2.15) | 0.07 |  |  |  |
| **(c) A_mass_–N_mass_ relationships** | | | | | |
| Asia | 1.42 (0.92, 2.19) | 0.36 | Asia | 1.77 (1.59, 1.97) | 0.65 |
| Australia | 1.89 (1.68, 2.13) | 0.14 | Australia | 1.35 (1.24, 1.46) | < 0.001 |
| Europe | 2.05 (1.84, 2.29) | < 0.01 | Europe | 2.30 (1.26, 4.22) | 0.42 |
| North America | 2.12 (1.84, 2.45) | < 0.01 | North America | 1.78 (1.63, 1.94) |  |
| South America | 3.22 (2.41, 4.30) | < 0.001 | South America | 1.37 (0.92, 2.02) | 0.23 |
| **(d) N_mass_–P_mass_ relationships** | | | | | |
| Africa | 0.97 (0.85, 1.10) | < 0.001 | Africa | 0.73 (0.67, 0.81) | < 0.05 |
| Asia | 0.73 (0.70, 0.78) | < 0.001 | Asia | 0.48 (0.45, 0.50) | <0.001 |
| Australia | 0.88 (0.81, 0.95) | < 0.001 | Australia | 0.63 (0.60, 0.66) | 0.07 |
| Europe | 0.78 (0.69, 0.88) | < 0.01 | Europe | 0.80 (0.77, 0.83) | < 0.001 |
| North America | 0.94 (0.73, 1.21) | < 0.01 | North America | 0.78 (0.69, 0.91) | < 0.05 |
| South America | 0.61 (0.51, 0.74) | 0.41 | South America | 0.47 (0.40, 0.54) | < 0.001 |

# Supplementary Reference

Adler, P. B., Milchunas, D. G., Lauenroth, W. K., Sala, O. E., and Burke, I. C. (2004). Functional traits of graminoids in semi-arid steppes: a test of grazing histories. *Journal of Applied Ecology* 41, 653–663. doi:10.1111/j.0021-8901.2004.00934.x.

Auger, S., and Shipley, B. (2013). Inter-specific and intra-specific trait variation along short environmental gradients in an old-growth temperate forest. *Journal of Vegetation Science* 24, 419–428. doi:10.1111/j.1654-1103.2012.01473.x.

Baruch, Z., and Goldstein, G. (1999). Leaf construction cost, nutrient concentration, and net CO2 assimilation of native and invasive species in Hawaii. *Oecologia* 121, 183–192. doi:10.1007/s004420050920.

Blonder, B., Baldwin, B. G., Enquist, B. J., and Robichaux, R. H. (2016). Variation and macroevolution in leaf functional traits in the Hawaiian silversword alliance (Asteraceae). *J. Ecol.* 104, 219–228. doi:10.1111/1365-2745.12497.

Boucher, F. C., Thuiller, W., Arnoldi, C., Albert, C. H., and Lavergne, S. (2013). Unravelling the architecture of functional variability in wild populations of Polygonum viviparum L. *Funct. Ecol.* 27, 382–391. doi:10.1111/1365-2435.12034.

Buchanan, S., Isaac, M. E., Meersche, K. V. den, and Martin, A. R. (2019). Functional traits of coffee along a shade and fertility gradient in coffee agroforestry systems. *Agroforest Syst* 93, 1261–1273. doi:10.1007/s10457-018-0239-1.

Butterfield, B. J., and Briggs, J. M. (2011). Regeneration niche differentiates functional strategies of desert woody plant species. *Oecologia* 165, 477–487. doi:10.1007/s00442-010-1741-y.

Campetella, G., Botta-Dukát, Z., Wellstein, C., Canullo, R., Gatto, S., Chelli, S., et al. (2011). Patterns of plant trait–environment relationships along a forest succession chronosequence. *Agriculture, Ecosystems & Environment* 145, 38–48. doi:10.1016/j.agee.2011.06.025.

Chacon-Madrigal, E., Wanek, W., Hietz, P., and Dullinger, S. (2018). Traits indicating a conservative resource strategy are weakly related to narrow range size in a group of neotropical trees. *Perspect. Plant Ecol. Evol. Syst.* 32, 30–37. doi:10.1016/j.ppees.2018.01.003.

Chen, Y., Han, W., Tang, L., Tang, Z., and Fang, J. (2013). Leaf nitrogen and phosphorus concentrations of woody plants differ in responses to climate, soil and plant growth form. *Ecography* 36, 178–184. doi:10.1111/j.1600-0587.2011.06833.x.

Cornelissen, J. H. C., Diez, P. C., and Hunt, R. (1996). Seedling growth, allocation and leaf attributes in a wide range of woody plant species and types. *J. Ecol.* 84, 755–765. doi:10.2307/2261337.

Craine, J. M., Elmore, A. J., Aidar, M. P. M., Bustamante, M., Dawson, T. E., Hobbie, E. A., et al. (2009). Global patterns of foliar nitrogen isotopes and their relationships with climate, mycorrhizal fungi, foliar nutrient concentrations, and nitrogen availability. *New Phytologist* 183, 980–992. doi:10.1111/j.1469-8137.2009.02917.x.

Craven, D., Braden, D., Ashton, M. S., Berlyn, G. P., Wishnie, M., and Dent, D. (2007). Between and within-site comparisons of structural and physiological characteristics and foliar nutrient content of 14 tree species at a wet, fertile site and a dry, infertile site in Panama. *For. Ecol. Manage.* 238, 335–346. doi:10.1016/j.foreco.2006.10.030.

Dahlin, K. M., Asner, G. P., and Field, C. B. (2013). Environmental and community controls on plant canopy chemistry in a Mediterranean-type ecosystem. *Proc. Natl. Acad. Sci. U. S. A.* 110, 6895–6900. doi:10.1073/pnas.1215513110.

Dalke, I. V., Novakovskiy, A. B., Maslova, S. P., and Dubrovskiy, Y. A. (2018). Morphological and functional traits of herbaceous plants with different functional types in the European Northeast. *Plant Ecol* 219, 1295–1305. doi:10.1007/s11258-018-0879-2.

Freschet, G. T., Cornelissen, J. H. C., van Logtestijn, R. S. P., and Aerts, R. (2010). Evidence of the “plant economics spectrum” in a subarctic flora. *Journal of Ecology* 98, 362–373. doi:10.1111/j.1365-2745.2009.01615.x.

Gos, P., Loucougaray, G., Colace, M.-P., Arnoldi, C., Gaucherand, S., Dumazel, D., et al. (2016). Relative contribution of soil, management and traits to co-variations of multiple ecosystem properties in grasslands. *Oecologia* 180, 1001–1013. doi:10.1007/s00442-016-3551-3.

Han, W., Chen, Y., Zhao, F.-J., Tang, L., Jiang, R., and Zhang, F. (2012). Floral, climatic and soil pH controls on leaf ash content in China’s terrestrial plants. *Glob. Ecol. Biogeogr.* 21, 376–382. doi:10.1111/j.1466-8238.2011.00677.x.

Herz, K., Dietz, S., Haider, S., Jandt, U., Scheel, D., and Bruelheide, H. (2017). Drivers of intraspecific trait variation of grass and forb species in German meadows and pastures. *J. Veg. Sci.* 28, 705–716. doi:10.1111/jvs.12534.

Joseph, G. S., Seymour, C. L., Cumming, G. S., Cumming, D. H. M., and Mahlangu, Z. (2014). Termite Mounds Increase Functional Diversity of Woody Plants in African Savannas. *Ecosystems* 17, 808–819. doi:10.1007/s10021-014-9761-9.

Kattenborn, T., Fassnacht, F. E., and Schmidtlein, S. (2019). Differentiating plant functional types using reflectance: which traits make the difference? *Remote Sens. Ecol. Conserv.* 5, 5–19. doi:10.1002/rse2.86.

Kattge, J., Knorr, W., Raddatz, T., and Wirth, C. (2009). Quantifying photosynthetic capacity and its relationship to leaf nitrogen content for global-scale terrestrial biosphere models. *Glob. Change Biol.* 15, 976–991. doi:10.1111/j.1365-2486.2008.01744.x.

Kichenin, E., Wardle, D. A., Peltzer, D. A., Morse, C. W., and Freschet, G. T. (2013). Contrasting effects of plant inter- and intraspecific variation on community-level trait measures along an environmental gradient. *Funct. Ecol.* 27, 1254–1261. doi:10.1111/1365-2435.12116.

La Pierre, K. J., and Smith, M. D. (2015). Functional trait expression of grassland species shift with short- and long-term nutrient additions. *Plant Ecol.* 216, 307–318. doi:10.1007/s11258-014-0438-4.

Laughlin, D. C., Fule, P. Z., Huffman, D. W., Crouse, J., and Laliberte, E. (2011). Climatic constraints on trait-based forest assembly. *J. Ecol.* 99, 1489–1499. doi:10.1111/j.1365-2745.2011.01885.x.

Lhotsky, B., Csecserits, A., Kovács, B., and Botta-Dukát, Z. (2016). New plant trait records of the hungarian flora. *Acta Botanica Hungarica* 59, 397–400.

Li, R., Zhu, S., Chen, H. Y. H., John, R., Zhou, G., Zhang, D., et al. (2015). Are functional traits a good predictor of global change impacts on tree species abundance dynamics in a subtropical forest? *Ecol. Lett.* 18, 1181–1189. doi:10.1111/ele.12497.

Li, Y., and Shipley, B. (2018). Community divergence and convergence along experimental gradients of stress and disturbance. *Ecology* 99, 775–781. doi:10.1002/ecy.2162.

Louault, F., Pillar, V. D., Aufrere, J., Garnier, E., and Soussana, J. F. (2005). Plant traits and functional types in response to reduced disturbance in a semi-natural grassland. *J. Veg. Sci.* 16, 151–160. doi:10.1111/j.1654-1103.2005.tb02350.x.

Lukes, P., Stenberg, P., Rautiainen, M., Mottus, M., and Vanhatalo, K. M. (2013). Optical properties of leaves and needles for boreal tree species in Europe. *Remote Sens. Lett.* 4, 667–676. doi:10.1080/2150704X.2013.782112.

Maire, V., Wright, I. J., Prentice, I. C., Batjes, N. H., Bhaskar, R., Bodegom, P. M. van, et al. (2015). Global effects of soil and climate on leaf photosynthetic traits and rates. *Global Ecology and Biogeography* 24, 706–717. doi:10.1111/geb.12296.

Martinez-Garza, C., Bongers, F., and Poorter, L. (2013). Are functional traits good predictors of species performance in restoration plantings in tropical abandoned pastures? *For. Ecol. Manage.* 303, 35–45. doi:10.1016/j.foreco.2013.03.046.

Messier, J., McGill, B. J., and Lechowicz, M. J. (2010). How do traits vary across ecological scales? A case for trait-based ecology. *Ecology Letters* 13, 838–848. doi:https://doi.org/10.1111/j.1461-0248.2010.01476.x.

Michaletz, S. T., and Johnson, E. A. (2006). A heat transfer model of crown scorch in forest fires. *Can. J. For. Res.-Rev. Can. Rech. For.* 36, 2839–2851. doi:10.1139/X06-158.

Miller, J. E. D., Ives, A. R., Harrison, S. P., and Damschen, E. I. (2018). Early- and late-flowering guilds respond differently to landscape spatial structure. *J. Ecol.* 106, 1033–1045. doi:10.1111/1365-2745.12849.

Montgomery, R. A., and Givnish, T. J. (2008). Adaptive radiation of photosynthetic physiology in the Hawaiian lobeliads: dynamic photosynthetic responses. *Oecologia* 155, 455–467. doi:10.1007/s00442-007-0936-3.

Ordoñez, J. C., van Bodegom, P. M., Witte, J.-P. M., Bartholomeus, R. P., van Hal, J. R., and Aerts, R. (2010). Plant Strategies in Relation to Resource Supply in Mesic to Wet Environments: Does Theory Mirror Nature? *Am. Nat.* 175, 225–239. doi:10.1086/649582.

Petter, G., Wagner, K., Wanek, W., Delgado, E. J. S., Zotz, G., Cabral, J. S., et al. (2016). Functional leaf traits of vascular epiphytes: vertical trends within the forest, intra- and interspecific trait variability, and taxonomic signals. *Functional Ecology* 30, 188–198. doi:10.1111/1365-2435.12490.

Powers, J. S., and Tiffin, P. (2010). Plant functional type classifications in tropical dry forests in Costa Rica: leaf habit versus taxonomic approaches. *Funct. Ecol.* 24, 927–936. doi:10.1111/j.1365-2435.2010.01701.x.

Prentice, I. C., Meng, T., Wang, H., Harrison, S. P., Ni, J., and Wang, G. (2011). Evidence of a universal scaling relationship for leaf CO2 drawdown along an aridity gradient. *New Phytol.* 190, 169–180. doi:10.1111/j.1469-8137.2010.03579.x.

Rolo, V., Lopez-Diaz, M. L., and Moreno, G. (2012). Shrubs affect soil nutrients availability with contrasting consequences for pasture understory and tree overstory production and nutrient status in Mediterranean grazed open woodlands. *Nutr. Cycl. Agroecosyst.* 93, 89–102. doi:10.1007/s10705-012-9502-4.

Seymour, C. L., Milewski, A. V., Mills, A. J., Joseph, G. S., Cumming, G. S., Cumming, D. H. M., et al. (2014). Do the large termite mounds of Macrotermes concentrate micronutrients in addition to macronutrients in nutrient-poor African savannas? *Soil Biol. Biochem.* 68, 95–105. doi:10.1016/j.soilbio.2013.09.022.

Sharpe, J. M., and N., S. (2016). Traits of fertile (spore-bearing) leaves of understory rainforest ferns from the El Verde Field Station in the El Yunque National Forest, Puerto Rico, USA. Unpublished data.

Shipley, B. (2002). Trade-offs between net assimilation rate and specific leaf area in determining relative growth rate: relationship with daily irradiance. *Funct. Ecol.* 16, 682–689. doi:10.1046/j.1365-2435.2002.00672.x.

Siefert, A., Fridley, J. D., and Ritchie, M. E. (2014). Community Functional Responses to Soil and Climate at Multiple Spatial Scales: When Does Intraspecific Variation Matter? *PLoS One* 9, e111189. doi:10.1371/journal.pone.0111189.

Smith, S. W., Woodin, S. J., Pakeman, R. J., Johnson, D., and van der Wal, R. (2014). Root traits predict decomposition across a landscape-scale grazing experiment. *New Phytol.* 203, 851–862. doi:10.1111/nph.12845.

Swaine, and E., K. (2007). Ecological and evolutionary drivers of plant community assembly in a Bornean rain forest. *University of Aberdeen*.

Swenson, N. G., Anglada-Cordero, P., and Barone, J. A. (2011). Deterministic tropical tree community turnover: evidence from patterns of functional beta diversity along an elevational gradient. *Proc. R. Soc. B-Biol. Sci.* 278, 877–884. doi:10.1098/rspb.2010.1369.

Takkis, K. (2014). Changes in plant species richness and population performance in response to habitat loss and fragmentation. *Dissertationes Biologicae Universitatis Tartuensis*, 255.

van de Weg, M. J., Meir, P., Grace, J., and Atkin, O. K. (2009). Altitudinal variation in leaf mass per unit area, leaf tissue density and foliar nitrogen and phosphorus content along an Amazon-Andes gradient in Peru. *Plant Ecol. Divers.* 2, 243-U7. doi:10.1080/17550870903518045.

van de Weg, M. J., Meir, P., Grace, J., and Damian Ramos, G. (2012). Photosynthetic parameters, dark respiration and leaf traits in the canopy of a Peruvian tropical montane cloud forest. *Oecologia* 168, 23–34. doi:10.1007/s00442-011-2068-z.

van der Plas, F., and Olff, H. (2014). Mesoherbivores affect grasshopper communities in a megaherbivore-dominated South African savannah. *Oecologia* 175, 639–649. doi:10.1007/s00442-014-2920-z.

Vergutz, L., Manzoni, S., Porporato, A., Novais, R. F., and Jackson, R. B. (2012). *A Global Database of Carbon and Nutrient Concentrations of Green and Senesced Leaves*. ORNL Distributed Active Archive Center doi:10.3334/ORNLDAAC/1106.

Walker, A. P., Aranda, I., Beckerman, A. P., Bown, H., Ce Rnusak, L. A., Dang, Q. L., et al. (2014). A Global Data Set of Leaf Photosynthetic Rates, Leaf N and P, and Specific Leaf Area. Data set. Available on-line [http://daac.ornl.gov] from Oak Ridge National Laboratory Distributed Active Archive Center, Oak Ridge, Tennessee, USA. http://dx.doi.org/10.3334/ORNLDAAC/1224.

Wang, H., Harrison, S. P., Prentice, I. C., Yang, Y., Bai, F., Furstenau Togashi, H., et al. (2017). *The China Plant Trait Database*. PANGAEA doi:10.1594/PANGAEA.871819.

Wright, I. J., Reich, P. B., Westoby, M., Ackerly, D. D., Baruch, Z., Bongers, F., et al. (2004). The worldwide leaf economics spectrum. *Nature* 428, 821–827. doi:10.1038/nature02403.

Wright, S. J., Kitajima, K., Kraft, N. J. B., Reich, P. B., Wright, I. J., Bunker, D. E., et al. (2010). Functional traits and the growth–mortality trade-off in tropical trees. *Ecology* 91, 3664–3674. doi:10.1890/09-2335.1.
